# Supplementary material for: Psychological Interventions and Bariatric Surgery among People with Clinically Severe Obesity—A Systematic Review with Bayesian Meta-Analysis
Source: Nutrients. 2022 Apr 12;14(8):1592. doi: 10.3390/nu14081592 (PMC9024573; doi:10.3390/nu14081592)

Psychological interventions and bariatric surgery among people with clinically severe obesity - a systematic review with Bayesian meta-analysis

A short title: Psychological interventions and bariatric surgery

Dawid Storman[1,2], Mateusz J Swierz[1,2], Monika Storman[2,3], Katarzyna W Jasinska[4], Pawel Jemiolo[5], Malgorzata M Bala[1,2]

[1]Department of Hygiene and Dietetics, Chair of Epidemiology and Preventive Medicine, Jagiellonian University Medical College, Krakow, Poland

[2] Systematic Reviews Unit, Jagiellonian University Medical College, Krakow, Poland

[3] Department of Diabetology and Internal Medicine, Medical University of Warsaw, Poland

[4]Students' Scientific Research Group, Systematic Reviews Unit – Polish Cochrane Branch, Jagiellonian University Medical College, Krakow, Poland

[5]AGH University of Science and Technology, al. Mickiewicza 30, 30-059, Krakow, Poland

Dawid Storman – dawid.storman@doctoral.uj.edu.pl

Mateusz J Swierz – mateusz.swierz@doctoral.uj.edu.pl

Monika Storman - monika.storman@wp.pl

Katarzyna W Jasinska - katarzyna.jasinska100@gmail.com

Pawel Jemiolo - pawljmlo@agh.edu.pl

Malgorzata M Bala - malgorzata.1.bala@uj.edu.pl

Corresponding author: Malgorzata M Bala

Chair of Epidemiology and Preventive Medicine, Jagiellonian University Medical College, Krakow, Poland

Address: Kopernika 7 31-034 Krakow, Poland

Phone: +48 12 422 37 20

e-mail: malgorzata.1.bala@uj.edu.pl

Appendices

Supplementary Materials, Table S1: The PRISMA checklist
Supplementary Materials, Table S2: Search strategies

Supplementary Materials, Table S3: List of ongoing studies

Supplementary Materials, Table S4: Included studies

Supplementary Materials, Table S5: List of excluded studies

Supplementary Materials, Figure S1: Graphical presentation of review authors' judgements about each risk of bias item for each included study

Supplementary Materials, Figure S2: Review authors' judgements about each risk of bias item presented as percentages across all included studies.
Supplementary Materials, Figure S3: Comparison between psychological intervention versus any control in the change of BMI at 6-12 months follow-up
Supplementary Materials, Figure S4: Comparison between psychological intervention versus any control in the change of BMI at 1-2 years follow-up
Supplementary Materials, Table S6: Results from random-effects meta-analyses
Supplementary Materials, Figure S5: Comparison between psychological intervention versus any control in the WL at 6-12 months follow-up
Supplementary Materials, Figure S6: Comparison between psychological intervention versus any control in the WL at 1-2 years follow-up
Supplementary Materials, Table S7: Psychosocial outcomes

Supplementary Materials, Figure S7: Subgroup analyses

Supplementary Materials, Table S1: The PRISMA checklist

Section/topic	#	Checklist item	Reported on page #
**TITLE**			
Title	1	Identify the report as a systematic review, meta-analysis, or both.	1
**ABSTRACT**			
Structured summary	2	Provide a structured summary including, as applicable: background; objectives; data sources; study eligibility criteria, participants, and interventions; study appraisal and synthesis methods; results; limitations; conclusions and implications of key findings; systematic review registration number.	3-5
**INTRODUCTION**			
Rationale	3	Describe the rationale for the review in the context of what is already known.	5-8
Objectives	4	Provide an explicit statement of questions being addressed with reference to participants, interventions, comparisons, outcomes, and study design (PICOS).	7-8
**METHODS**			
Protocol and registration	5	Indicate if a review protocol exists, if and where it can be accessed (e.g., Web address), and, if available, provide registration information including registration number.	8
Eligibility criteria	6	Specify study characteristics (e.g., PICOS, length of follow-up) and report characteristics (e.g., years considered, language, publication status) used as criteria for eligibility, giving rationale.	8-10

Information sources	7	Describe all information sources (e.g., databases with dates of coverage, contact with study authors to identify additional studies) in the search and date last searched.	10
Search	8	Present full electronic search strategy for at least one database, including any limits used, such that it could be repeated.	Supplementary Materials A
Study selection	9	State the process for selecting studies (i.e., screening, eligibility, included in systematic review, and, if applicable, included in the meta-analysis).	10-11
Data collection process	10	Describe method of data extraction from reports (e.g., piloted forms, independently, in duplicate) and any processes for obtaining and confirming data from investigators.	11
Data items	11	List and define all variables for which data were sought (e.g., PICOS, funding sources) and any assumptions and simplifications made.	11
Risk of bias in individual studies	12	Describe methods used for assessing risk of bias of individual studies (including specification of whether this was done at the study or outcome level), and how this information is to be used in any data synthesis.	11-12
Summary measures	13	State the principal summary measures (e.g., risk ratio, difference in means).	12-13
Synthesis of results	14	Describe the methods of handling data and combining results of studies, if done, including measures of consistency (e.g., I^2) for each meta-analysis.	12
Section/topic	#	Checklist item	Reported on page #
Risk of bias across studies	15	Specify any assessment of risk of bias that may affect the cumulative evidence (e.g., publication bias, selective reporting within studies).	13
Additional analyses	16	Describe methods of additional analyses (e.g., sensitivity or subgroup analyses, meta-regression), if done, indicating which were pre-specified.	13
**RESULTS**			
Study selection	17	Give numbers of studies screened, assessed for eligibility, and included in the review, with reasons for exclusions at each stage, ideally with a flow diagram.	13, Supplementary Materials D, Figure 1
Study characteristics	18	For each study, present characteristics for which data were extracted (e.g., study size, PICOS, follow-up period) and provide the citations.	Table 1, Supplementary Materials C
Risk of bias within studies	19	Present data on risk of bias of each study and, if available, any outcome level assessment (see item 12).	Supplementary Materials C
Results of individual studies	20	For all outcomes considered (benefits or harms), present, for each study: (a) simple summary data for each intervention group (b) effect estimates and confidence intervals, ideally with a forest plot.	Table 2,
Synthesis of results	21	Present results of each meta-analysis done, including confidence intervals and measures of consistency.	Table 3, Supplementary Materials F,G

Risk of bias across studies	22	Present results of any assessment of risk of bias across studies (see Item 15).	Figure 2, Supplementary Materials E
Additional analysis	23	Give results of additional analyses, if done (e.g., sensitivity or subgroup analyses, meta-regression [see Item 16]).	Supplementary Materials H
**DISCUSSION**			
Summary of evidence	24	Summarize the main findings including the strength of evidence for each main outcome; consider their relevance to key groups (e.g., healthcare providers, users, and policy makers).	22
Limitations	25	Discuss limitations at study and outcome level (e.g., risk of bias), and at review-level (e.g., incomplete retrieval of identified research, reporting bias).	23
Conclusions	26	Provide a general interpretation of the results in the context of other evidence, and implications for future research.	25-26
**FUNDING**			
Funding	27	Describe sources of funding for the systematic review and other support (e.g., supply of data); role of funders for the systematic review.	2

**Supplementary Materials, Table S2: Search strategies**
**1 MEDLINE search strategy**
1. exp obesity/
2. Overweight/
3. over?weight.ti,ab.
4. over weight.ti,ab.
5. overeating.ti,ab.
6. over?eating.ti,ab.
7. or/1-6
8. exp Weight Loss/
9. weight loss.ti,ab.
10. weight reduc$.ti,ab.
11. Weight loss surg$.ti.ab.
12. Weight reduc surg$.ti.ab.
13. bariatric surg$.ti,ab.
14. exp bariatric surgery/
15. (surg$ adj5 bariatric).ti,ab.
16. anti?obesity surg$.ti,ab.
17. antiobesity surg$.ti,ab.

18. (obesity adj5 surgery).ti,ab.
19. (obesity adj5 surgical).ti,ab.
20. (gastroplasty or gastro?gastostomy or "gastric bypass" or "gastric surgery" or "restrictive surgery").ti,ab.
21. exp gastric bypass/
22. exp jejunoileal bypass/
23. jejuno?ileal bypass.ti,ab.
24. jejunoileal bypass.ti,ab.
25. gastrointestinal surg$.ti,ab.
26. gastrointestinal diversion$.ti,ab.
27. exp biliopancreatic diversion/
28. biliopancreatic diversion.ti,ab.
29. bilio?pancreatic diversion.ti,ab.
30. biliopancreatic bypass.ti,ab.
31. bilio?pancreatic bypass.ti,ab.
32. gastric band$.ti,ab.
33. silicon band$.ti,ab.
34. exp gastroenterostomy/
35. gastrectomy.ti,ab.
36. gastroplasty/
37. LAGB.ti,ab.
38. stomach stapl$.ti,ab.
39. lap band$.ti,ab.
40. lap-band$.ti,ab.
41. malabsorptive surg$.ti,ab.
42. mason$ procedure.ti,ab.
43. "Roux-en-Y".ti,ab.
44. anastomosis, Roux-en-Y/
45. malabsorptive procedure$.ti,ab.
46. duodenal switch$.ti,ab.
47. obesity/su
48. exp Obesity, Morbid/su [Surgery]
49. or/8-48
50. psychotherapy.mp. or exp Psychotherapy/ or exp Psychotherapy, Group/ or psychotherapeutic*.mp. or Psychotherapy/
51. ((psycho* or behav*) adj3 (therap* or treatment* or intervention* or counsel* or support*)).tw.
52. supportive expressive group therapy.mp.

53. group support*.mp.
54. group intervention*.mp.
55. exp Self-Help Groups/ or self-help group*.mp.
56. exp Counseling/ or counsel.mp.
57. counsel*.ti,ab.
58. expressive writing.mp.
59. ((behavio?r* or motivation*) adj3 (activation or therap* or treatment* or intervention* or modification* or contract* or program* or counsel*)).tw.
60. (cognitiv* adj3 (therap* or treatment* or intervention* or control* or program*)).tw.
61. exp Cognitive Therapy/ or cognitive behavioural therapy.mp.
62. (train* adj3 (autogenic or assertive* or mind or sensitivity or relax*)).tw.
63. or/50-62
64. 7 and 49 and 63
65. randomized controlled trial.pt.
66. controlled clinical trial.pt.
67. randomized.ab.
68. placebo.ab.
69. drug therapy.fs.
70. randomly.ab.
71. trial.ab.
72. groups.iab.
73. or/65-72
74. Exp animals/ NOT humans.sh
75. 73 NOT 74
76. 64 and 75
**2 Embase search strategy**
1 exp OBESITY/ or exp MORBID OBESITY/
2 over?weight.ti,ab.
3 over weight.ti,ab.
4 overeating.ti,ab.
5 over?eating.ti,ab.
6 or/1-5
7 exp Weight Reduction/
8 (weight adj1 los*).ti,ab.
9 (weight adj1 loos*).ti,ab.
10 weightloss.ti,ab.

11 weight?loss.ti,ab.
12 (weight adj3 reduc*).ti,ab.
13 weight?reduc*.ti,ab.
14 "bariatric surg*".ti,ab.
15 exp Bariatric Surgery/
16 (surg* adj5 bariatric).ti,ab.
17 (anti?obesity adj3 surg*).ti,ab.
18 (antiobesity adj3 surg*).ti,ab.
19 anti obesity surg*.ti,ab.
20 (obesity adj5 surgery).ti,ab.
21 (obesity adj5 surgical).ti,ab.
22 (gastroplasty or gastrogastrostomy or gastro?gastrostomy or gastroenterostomy or "gastric bypass" or "gastric surgery" or "restrictive surgery").ti,ab.
23 exp Stomach Bypass/
24 exp Jejunoileal Bypass/
25 jejuno?ileal bypass.ti,ab.
26 jejunoileal bypass.ti,ab.
27 gastrointestinal surg*.ti,ab.
28 gastrointestinal diversion*.ti,ab.
29 (gastro-intestinal adj5 diversion).ti,ab.
30 exp Biliopancreatic Bypass/
31 Biliopancreatic Bypass.ti,ab.
32 Biliopancreatic diversion.ti,ab.
33 bilio?pancreatic diversion.ti,ab.
34 bilio?pancreatic bypass.ti,ab.
35 gastric band*.ti,ab.
36 exp Gastric Banding/
37 silicon band*.ti,ab.
38 exp GASTROENTEROSTOMY/
39 gastroenterostomy.ti,ab.
40 exp GASTRECTOMY/
41 gastrectomy.ti,ab.
42 exp GASTROPLASTY/
43 LAGB.ti,ab.
44 stomach stapl*.ti,ab.
45 gastric stapl*.ti,ab.

46 lap band*.ti,ab.
47 lap-band*.ti,ab.
48 malabsorptive surg*.ti,ab.
49 mason* procedure.ti,ab.
50 "roux-en-Y".ti,ab.
51 exp Roux y Anastomosis/
52 malabsorpti* procedure*.ti,ab.
53 malabsorpti* surg*.ti,ab.
54 duodenal switch*.ti,ab.
55 or/7-54
56 6 and 55
57 OBESITY/su [Surgery]
58 Morbid Obesity/su [Surgery]
59 57 or 58
60 6 and 59
61 56 or 60
62 psychotherapy.mp. or exp Psychotherapy/ or exp Psychotherapy, Group/
63 supportive expressive group therapy.mp.
64 exp Cognitive Therapy/ or cognitive behavioural therapy.mp.
65 group support*.mp.
66 group intervention*.mp.
67 exp Self-Help Groups/ or self-help group*.mp.
68 Counseling/ or counsel.mp.
69 counsel*.ti,ab.
70 expressive writing.mp.
71 psychotherapeutic*.mp. or Psychotherapy/
72 adaptation, psychological/
73 or/62-72
74 61 and 73
75 Randomized Controlled Trial/
76 Randomization/
77 Single Blind Procedure/
78 Double Blind Procedure/
79 ((single or doubl* or trebl* or tripl*) adj (mask* or blind*)).tw.
80 (placebo* and control* and trial*).tw.

81 randomi?ed control* trial*.tw.
82 (random* adj2 allocat*).tw.
83 (placebo* and random* and (trial* or study or studies)).tw.
84 (randomized or randomised).tw.
85 Controlled Clinical Trial/
86 or/75-85
87 74 AND 86
**3 The Cochrane Library search strategy**
#1 MeSH descriptor: [Obesity] explode all trees
#2 MeSH descriptor: [Overweight] this term only
#3 (obes* or overweight or "over weight")
#4 #1 or #2 or #3
#5 MeSH descriptor: [Weight Loss] explode all trees
#6 MeSH descriptor: [Bariatric Surgery] explode all trees
#7 (bariatric near/5 surg*)
#8 (obes* near/5 surg*)
#9 ((antiobesity or anti-obesity or "anti obesity") near/5 (surg*))
#10(gastroplasty or gastrogastrostomy or gastro?gastrostomy or gastroenterostomy or "gastric bypass" or "gastric surgery" or "restrictive surgery")
#11 MeSH descriptor: [Gastric Bypass] explode all trees
#12 MeSH descriptor: [Jejunoileal Bypass] explode all trees
#13 ((jejunoileal or "jejuno-ilial" or "jejuno ilial") next (bypass))
#14 gastrointestinal next surg*
#15 gastrointestinal next diversion*
#16 biliopancreatic diversion
#17 MeSH descriptor: [Biliopancreatic Diversion]
#18 "gastric band*"
#19 "silicon band*"
#20 MeSH descriptor: [Gastroenterostomy] explode all trees
#21 gastrectomy
#22 MeSH descriptor: [Gastroplasty] explode all trees
#23 LAGB:ti,ab
#24 stomach near/5 stapl*
#25 gastric near/5 stapl*
#26 lap next band*
#27 mason* next procedure

#28 "roux-en-Y"
#29 MeSH descriptor: [Anastomosis, Roux-en-Y] explode all trees
#30 malabsorpti* next procedure*
#31 malabsorpti* next surg*
#32 duodenal next switch*
#33 #5 or #6 or #7 or #8 or #9 or #10 or #11 or #12 or #13 or #14 or #15 or #16 or #17 or #18 or #19 or #20 or #21 or #22 or #23 or #24 or #25 or #26 or #27 or #28 or #29 or #30 or #31 or #32
#34 MeSH descriptor: [Psychotherapy] explode all trees
#35 MeSH descriptor: [Psychotherapy, Group] explode all trees
#36 MeSH descriptor: [Social Support] explode all trees
#37 MeSH descriptor: [Cognitive Therapy] explode all trees
#38 MeSH descriptor: [Behavior Therapy] explode all trees
#39 MeSH descriptor: [Cognitive Therapy] explode all trees
#40 MeSH descriptor: [Counseling] explode all trees
#41 psychotherapeutic or CBT or acceptance and commitment therapy or psycho-educational intervention
#42 ((psycho* or behav*) near/3 (therap* or treatment* or intervention* or counsel* or support*)):ti,ab,kw
#43 ((behavior* or behaviour* or motivation*) near/3 (activation or therap* or treatment* or intervention* or modification* or contract* or program* or counsel*)):ti,ab,kw
#44 (cognitiv* near/3 (therap* or treatment* or intervention* or control* or program*)):ti,ab,kw
#45 (train* near/3 (autogenic or assertive* or mind or sensitivity or relax*)):ti,ab,kw
#46 #34 or #35 or #36 or #37 or #38 or #38 or #39 or #40
#47 #4 and #33 and #46
**4 ClinicalTrials**
obesity AND psychological AND EXACT "Interventional" [STUDY-TYPES]
obesity AND psychology AND EXACT "Interventional" [STUDY-TYPES]
obesity AND psychosocial AND EXACT "Interventional" [STUDY-TYPES]
obesity AND psychotherapy AND EXACT "Interventional" [STUDY-TYPES]
obesity AND psychotherapeutic AND EXACT "Interventional" [STUDY-TYPES]
obesity AND cbt
obesity AND cognitive behavioral therapy
overweight AND psychological AND EXACT "Interventional" [STUDY-TYPES]
overweight AND psychology AND EXACT "Interventional" [STUDY-TYPES]
overweight AND psychosocial AND EXACT "Interventional" [STUDY-TYPES]
overweight AND psychotherapy AND EXACT "Interventional" [STUDY-TYPES]
overweight AND psychotherapeutic AND EXACT "Interventional" [STUDY-TYPES]
overweight AND cbt
overweight AND cognitive behavioral therapy

overeating AND psychological AND EXACT "Interventional" [STUDY-TYPES]
overeating AND psychology AND EXACT "Interventional" [STUDY-TYPES]
overeating AND psychosocial AND EXACT "Interventional" [STUDY-TYPES]
overeating AND psychotherapy AND EXACT "Interventional" [STUDY-TYPES]
overeating AND psychotherapeutic AND EXACT "Interventional" [STUDY-TYPES]
overeating AND cbt
overeating AND cognitive behavioral therapy

Supplementary Materials, Table S3: List of ongoing studies
**American 2012**<br>American University of Sharfah, Sheikh Khalifa Medical City, Jordan Hospital. Obesity Surgery, Counselling, and Psychological Well-Being. 2012.
**Conceição 2016**<br>Conceição E., Machado P., Vaz A., Pinto-Bastos A., Ramalho S., Silva C., et al. 2016. APOLO-Bari, an internet-based program for longitudinal support of bariatric surgery patients: study protocol for a randomized controlled trial. 17(1):114.
**Geisinger 2017**<br>Geisinger Clinic. Pilot Behavioral Support Intervention After Bariatric Surgery. 2017.
**Mangieri 2017**<br>Mangieri C., Johnson R., Choi Y., Wood J., Eisenhower D. 2017. Mobile health applications, do they enhance weight loss efficacy following bariatric surgery. 31, S7.
**Mayo 2009**<br>Mayo Clinic. 2009. Behavior Therapy Prior to Bariatric Surgery. https://ClinicalTrials.gov/show/NCT01125397
**National 2016**<br>National University of Ireland Galway Ireland. 2016. Compassion-Focused Therapy for People With Severe Obesity. https://ClinicalTrials.gov/show/NCT03249441
**Owers 2017a**<br>Owers C., Ackroyd R., Halliday V. 2017. Can structured psychosocial eDUCATIon improve health related quality of life outcomes following bariatric surgery?: A pilot study pre-operative management. Obesity Surgery, 27, 831.
**Owers 2017b**<br>Owers C., Halliday V., Ackroyd R. 2017. The effect of improved pre-operative education on the health-related quality of life outcomes following bariatric surgery dragons' den meets shark tank (proposals for randomized controlled trials). Obesity Surgery, 27, 249-50.
**Paul 2015**<br>Paul L., Rongen S., Hoeken D., Deen M., Klaassen R., Biter L., et al. 2015. Does cognitive behavioral therapy strengthen the effect of bariatric surgery for obesity? Design and methods of a randomized and controlled study. 42, 252-6.
**Sellberg 2018**<br>*[DOI: 10.1186/s12893-018-0358-7; ISRCTN: 16417174]*<br>Sellberg F, Possmark S, Ghaderi A, Naslund E, Willmer M, Tynelius P et al. 2018. A dissonance-based intervention for women post roux-en-Y gastric bypass surgery aiming at improving quality of life and physical activity 24 months after surgery: study protocol for a randomized controlled trial. BMC surgery, 18, 25.
**Sheffield 2013**<br>Sheffield Teaching Hospitals NHS Foundation Trust. 2013. Psychological Preparation Prior to Bariatric Surgery. https://ClinicalTrials.gov/show/NCT01670331.
**Thakker 2017**<br>Thakker F., Khamkar A., Shah V., Shah S., Shah P. 2017. Digital support group (DSG) better than actual in postoperative management after bariatric surgery Post-operative care. Obesity Surgery, 27, 193.
**University 2013**<br>University Hospital Grenoble. 2013. Mindfulness in Preparation to Bariatric Surgery. https://ClinicalTrials.gov/show/NCT02854306.
**University 2013a**<br>University of Surrey, University College London Hospitals. 2013. The Impact of an Investment Based Intervention on Weight-loss and Beliefs About Food in Patients Post Bariatric Surgery. https://ClinicalTrials.gov/show/NCT02045628.
**University 2017**<br>University College London. 2017. Evaluation of a Lifestyle Intervention After Bariatric Surgery. https://ClinicalTrials.gov/show/NCT03214471.
**Yale 2014**<br>Yale University. 2014. Loss of Control Eating Following Weight Loss Surgery. https://ClinicalTrials.gov/show/NCT02259322.

Supplementary Materials, Table S4: Included studies	
**Hjelmesæth 2018**	*[ClinicalTrials.gov: NCT01403558]*<br>Gade H., Friborg O., Rosenvinge J., Småstuen M., Hjelmesæth J. 2015. The Impact of a Preoperative Cognitive Behavioural Therapy (CBT) on Dysfunctional Eating Behaviours, Affective Symptoms and Body Weight 1 Year after Bariatric Surgery: A Randomised Controlled Trial. Obesity surgery, 25(11), 2112-9.<br>Gade H., Friborg O., Sandbu R., Rosenvinge J., Hjelmesaeth J. 2017. Long-term follow-up (4 years) of patients receiving cognitive behavioural therapy (CBT) or usual care before bariatric surgery: A randomized controlled trial. Obesity Facts, 10(1), 38.<br>Gade H., Hjelmesæth J., Rosenvinge J. H., Friborg O. 2014. Effectiveness of a cognitive behavioral therapy for dysfunctional eating among patients admitted for bariatric surgery: a randomized controlled trial. Journal of Obesity, 2014, 1-6. [DOI: 10.1155/2014/127936; Other: NCT01403558]<br>Gade H., Rosenvinge J., Friborg O., Hjelmesaeth J. 2013. Cognitive behavioural therapy significantly reduces emotional eating in bariatric surgery patients. A randomised controlled trial. Obesity facts, 6, 30-1.<br>Gade H., Rosenvinge J. H., Hjelmesæth J., Friborg O. 2014. Psychological correlates to dysfunctional eating patterns among morbidly obese patients accepted for bariatric surgery. Obesity Facts, 7(2), 111-9.<br>Hjelmesæth J., Rosenvinge J. H., Gade H., Friborg O. 2018. Effects of cognitive behavioral therapy on eating behaviors, affective symptoms, and weight loss after bariatric surgery: a randomized clinical trial. Obesity Surgery. [DOI: 10.1007/s11695-018-3471-x; Other: NCT01403558]
**Hollywood 2015**	*[ClinicalTrials.gov: NCT01264120]*<br>Hollywood A., Ogden J., Pring C. 2015. The Impact of Psychological Support on Psychological Outcomes and Patients' Experiences of the Bariatric Service 1 and 2 Years after Bariatric Surgery. Journal of Obesity and Bariatrics, 2(1), 1-7.<br>Hollywood A., Ogden J., Pring C. 2012. The impact of a bariatric rehabilitation service on weight loss and psychological adjustment - study protocol. BMC Public Health, 12(1), 275.<br>Ogden J., Hollywood A., Pring C. 2015. The impact of psychological support on weight loss post weight loss surgery: a randomised control trial. Obesity Surgery, 25(3), 500-5.<br>University of Surrey, Western Sussex Hospitals NHS Trust, National Institute for Health Research United Kingdom. 2011. The impact of a bariatric rehabilitation service on patient outcomes. https://clinicaltrials.gov/ct2/show/NCT01264120.
**Kalarchian 2012**	Kalarchian M., Marcus M., Courcoulas A., Cheng Y., Levine M., et al. 2012. Optimizing long-term weight control after bariatric surgery: a pilot study. Surgery for Obesity and Related Diseases, 8(6), 710-5.
**Kalarchian 2016**	*[ClinicalTrials.gov: NCT00623792]*<br>Kalarchian M., Marcus M., Courcoulas A., Cheng Y., Levine M. 2016. Preoperative lifestyle intervention in bariatric surgery: a randomized clinical trial. Surgery for Obesity and Related Diseases, 12(1), 180-7.<br>Kalarchian M., Marcus M., Courcoulas A., Cheng Y., Levine M. 2013. Preoperative lifestyle intervention in bariatric surgery: initial results from a randomized, controlled trial. Obesity, 21(2), 254-60.<br>Kalarchian M. A., Marcus M. D., Courcoulas A. P., Cheng Y., Levine M. D.2014. Self-report of gastrointestinal side effects after bariatric surgery. Surgery for Obesity and Related Diseases, 10(6), 1202-7.
**Lier 2012**	*[ClinicalTrials.gov: NCT00635011]*<br>Helse F., Helse V. 2008. Pre- and postoperative cognitive behavior therapy for patients accepted for bariatric surgery. https://clinicaltrials.gov/ct2/show/NCT00635011.<br>Lier H. Ø., Biringer E., Eriksen H., Tangen T. 2009. Subjective health complaints in a sample with morbid obesity and the complaints' relation with work ability. European Psychiatry, 24(P02-60), S750.<br>Lier H. Ø., Biringer E., Hove O., Stubhaug B., Tangen T. 2011. Quality of life among patients undergoing bariatric surgery: associations with mental health- A 1 year follow-up study of bariatric surgery patients. Health and Quality of Life Outcomes, 9(1), 79-89.<br>Lier H. Ø., Biringer E., Stubhaug B., Eriksen H. R., Tangen T. 2010. Psychiatric disorders and participation in pre- and postoperative counselling groups in bariatric surgery patients. Obesity Surgery, 21(6), 730-7.<br>Lier H. Ø., Biringer E., Stubhaug B., Tangen T. 2012. The impact of preoperative counseling on postoperative treatment adherence in bariatric surgery patients: a randomized controlled trial. Patient education and counselling, 87(3), 336-42.<br>Lier H., Biringer E., Stubhaug B., Eriksen H. R., Tangen T. 2010. Patient outcome expectancy from bariatric surgery. European Psychiatry, 25(P02-195), 819.
**Paul 2021**	Paul, L., van der Heiden, C., van Hoeken, D., Deen, M., Vlijm, A., Klaassen, R. A., ... & Hoek, H. W. (2021). Cognitive Behavioral Therapy Versus Usual Care Before Bariatric Surgery: One-Year Follow-Up Results of a Randomized Controlled Trial. Obesity Surgery, 31(3), 970-979.
**Petasne 2013**	Petasne Nijamkin M., Campa A., Samiri Nijamkin S., Sosa J. 2013. Comprehensive behavioral-motivational nutrition education improves depressive symptoms following bariatric surgery: a randomized, controlled trial of obese Hispanic Americans. Journal of Nutrition Education and Behavior, 45(6), 620-6.<br>Petasne Nijamkin M., Campa A., Sosa J., Baum M., Himburg S., Johnson P. 2012. Comprehensive nutrition and lifestyle education improves weight loss and physical activity in Hispanic Americans following gastric bypass surgery: a randomized controlled trial. Journal of the Academy of Nutrition and Dietetics, 112(3), 382-90.
**Tucker 1991**	Tucker J., Samo J., Rand C., Woodward E. 1991. Behavioral interventions to promote adaptive eating behavior and lifestyle changes following surgery for obesity: results of a Two-year outcome evaluation. International Journal of Eating Disorders, 10(6), 689-98.
**Wild 2017**	*[ISRCTN: 11076390]*<br>Wild B., Hünnemeyer K., Sauer H., Hain B., Mack I., et al. 2015. A 1-year videoconferencing-based psychoeducational group intervention following bariatric surgery: results of a randomized controlled study. Surgery for Obesity and Related Diseases, 11(6), 1349-60.<br>Wild B., Hünnemeyer K., Sauer H., Schellberg D., Müller-Stich B. P., et al. 2017. Sustained effects of a psychoeducational group intervention following bariatric surgery: Follow-up of the randomized controlled BaSE study. Surgery for Obesity and Related Diseases, 13(9), 1612-8.<br>Wild B., Hünnemeyer K., Sauer H., Teufel M. 2017. Psychotherapeutic interventions following bariatric surgery. Implications from the BaSE study [Psychotherapeutische Interventionen nach Adipositaschirurgie]. Psychotherapeut, 62(3), 222-9.

Hjelmesæth 2018

**Methods**	RCT, parallel assignment, open label, block randomization, with blocks of 4, the allocation ratio was 1:1, descriptive statistics; t-test; chi-square tests; Linear mixed model regression<br><br>Country: Norway<br><br>Settings: Senter for sykelig overvekt; Helse Sør-Øst, Sykehuset i Vestfold<br><br>Number of centers: 1<br><br>COI: None of the authors has any potential financial conflict of interest related to the manuscript<br><br>Funding: The first author has been funded by an unrestricted research grant from the South-Eastern Norway Regional Health Authority<br><br>Protocol registration: Clinicaltrials.gov: NCT01403558
**Participants**	Inclusion Criteria: being accepted for BS<br><br>Exclusion Criteria: suffering from drug and/or alcohol addiction.<br><br>Number of participants screened: 167<br><br>Number of participants who met inc&exc criteria: Not reported<br><br>Number of patients randomized: Intervention: 50, Control: 52<br><br>Number of patients who underwent bariatric surgery: Intervention: 42, Control: 38<br><br>Type of bariatric surgery: Intervention: RYGB 36 (85.7%); Sleeve gastrectomy 6 (14.3%) , Control: RYGB 31 (81.6%); Sleeve gastrectomy 7 (18.4%)<br><br>Number of patients analysed at baseline: Intervention: 42, Control: 38<br><br>Age mean (SD): Intervention: 44.1 (9.8), Control: 41.2 (9.6)<br><br>Female N (%): Intervention: 27 (64.3%), Control: 28 (73.7%)<br><br>Comorbidities N (%): Not reported
**Interventions**	**Intervention:** PreOP CBT for 10weeks<br><br>**Control:** 10 weeks of nutritional support and education
**Outcomes**	Primary Outcome<br><br>- Changes in eating behaviour and affective symptoms after psychological intervention<br><br>- Eating behaviours
**Notes**	Tertiary care centre in Norway, September 2011 and December 2013
BS – bariatric surgery; CBT, Cognitive-behavioral therapy; COI - Conflict of interest; OP – operation; RCT – randomized controlled trials, RYGB, Roux-en-Y gastric bypass; SD - Standard deviation	

Risk of bias table

Bias	Authors' judgement	Support for judgement
Random sequence generation (selection bias)	Low risk	Block randomisation with block size of 4; Two research assistants at the treatment centre with no affiliation to the study had access to the randomisation file.
Allocation concealment (selection bias)	Low risk	Two research assistants at the treatment centre with no affiliation to the study had access to the randomisation file
Blinding of participants and personnel (performance bias) Weight loss/BMI	Low risk	Objective outcome, no blinding, but the review authors judge that the outcome was not likely to be influenced by lack of blinding
Blinding of participants and personnel (performance bias) Psychological outcomes	High risk	Filled out by patients, open study, outcome could be influenced
Blinding of participants and personnel (performance bias) Nonnormative eating pattern	High risk	Filled out by patients, open study, outcome could be influenced
Blinding of outcome assessment (detection bias) Weight loss/BMI	Low risk	Objective outcome, no blinding, but the review authors judge that the outcome was not likely to be influenced by lack of blinding
Blinding of outcome assessment (detection bias) Psychological outcomes	High risk	Filled out by patients, open study, outcome could be influenced
Blinding of outcome assessment (detection bias) Nonnormative eating pattern	High risk	Filled out by patients, open study, outcome could be influenced
Incomplete outcome data (attrition bias)	High risk	Reasons for drop-out reported but unclear why not all patients accepted invitation for 1-year follow-up - the characteristics of the subgroup of patients who did not accept invitation was not compared to those who accepted invitation, patients excluded after randomisation, imbalance in numbers of missing patients, unclear characteristics of the subgroup
Selective reporting (reporting bias)	Low risk	Outcomes reported as defined in protocol.
Other bias	Low risk	None identified
BMI – body mass index		

Hollywood 2015

**Methods**	RCT, parallel assignment, open label<br><br>Country: United Kingdom<br><br>Settings: St. Richard's Hospital in Chichester, West Sussex<br><br>Number of centers: 1<br><br>COI: The authors have no conflict of interest to declare<br><br>Funding: Funded by RfPB NIHR, Grant number: PBPG-0909-20178. This trial was conducted and data collected with complete independence of the researchers from the RfPB NIHR<br><br>Protocol registration: Clinicaltrials.gov: NCT01264120
**Participants**	Inclusion Criteria: if they consent, >=18yrs, attended the bariatric clinic at Chichester, accepted for BS, able to pay for BS<br><br>Exclusion Criteria: no wish to have BS because of not being a suitable patient/able to secure funding from the primary care trust for the surgery, no English skills.<br><br>Number of participants screened: 206<br><br>Number of participants who met inc&exc criteria: Not reported<br><br>Number of patients randomized: Intervention: 82, Control: 80<br><br>Number of patients who underwent bariatric surgery: Intervention: 82, Control: 80<br><br>Type of bariatric surgery: RYGB (100%)<br><br>Number of patients analysed at baseline: Intervention: 82, Control: 80<br><br>Age mean (SD): Intervention: 45.6 (11.1), Control: 44.8 (10.6)<br><br>Female N (%): Intervention: 61 (74.4%), Control: 61 (76.2%)<br><br>Comorbidities N (%): Not reported
**Interventions**	**Intervention:** Health psychology-led BRS. CAU+ 3 one-to-one 50-min sessions with a health psychologist 2 weeks preOP, postOP and at 3 mo follow-up.<br><br>**CAU:** preOP tests and a standard diet sheet postOP
**Outcomes**	Primary Outcome:<br><br>- Change in weight and BMI<br>Secondary Outcome<br><br>- Change in psychological measures (QoL, dietary control, coping strategies and emotional eating)<br>- Cost-effectiveness
BRS - bariatric rehabilitation service; BS – bariatric surgery; CAU - care as usual; CBT - Cognitive-behavioral therapy; COI - Conflict of interest; OP – operation; QoL - Quality of life; RCT – randomized controlled trials, RYGB - Roux-en-Y gastric bypass; SD - Standard deviation	

Risk of bias table

Bias	Authors' judgement	Support for judgement
Random sequence generation (selection bias)	Unclear risk	No information in publication nor in protocol
Allocation concealment (selection bias)	Low risk	Third-party-blinded randomization
Blinding of participants and personnel (performance bias)<br>Weight loss/BMI	Unclear risk	Objective outcome, assessed in the clinic 2 weeks pre and 12 weeks post - impossible to blind a patient throughout the whole study as he could guess from sessions format in which group he was placed
Blinding of participants and personnel (performance bias)<br>Quality of life	High risk	Filled out by patients, open study, outcome could be influenced
Blinding of participants and personnel (performance bias)<br>Psychological outcomes	High risk	Filled out by patients, open study, outcome could be influenced
Blinding of participants and personnel (performance bias)<br>Nonnormative eating pattern	High risk	Filled out by patients, open study, outcome could be influenced
Blinding of outcome assessment (detection bias)<br>Weight loss/BMI	Unclear risk	Objective outcome, assessed in the clinic 2 weeks pre and 12 weeks post - impossible to blind a patient throughout the whole study as he could guess from sessions format in which group he was placed
Blinding of outcome assessment (detection bias)<br>Quality of life	High risk	Filled out by patients, open study, outcome could be influenced
Blinding of outcome assessment (detection bias)<br>Psychological outcomes	High risk	Filled out by patients, open study, outcome could be influenced
Blinding of outcome assessment (detection bias)<br>Nonnormative eating pattern	High risk	Filled out by patients, open study, outcome could be influenced
Incomplete outcome data (attrition bias)	High risk	Very low response rate for questionnaires concerning psychological assessment: response rate=43.2%
Selective reporting (reporting bias)	Low risk	Outcomes reported as stated in protocol
Other bias	Low risk	None identified
BMI – body mass index		

Kalarchian 2012

**Methods**	RCT, the Wilcoxon rank sum test, Fisher's exact test, Spearman's correlation<br><br>Country: United States of America<br><br>Settings: Not reported<br><br>Number of centers: Not reported<br><br>COI: The authors have no commercial associations that might be a conflict of interest in relation to this article<br><br>Funding: A 2007 Research Grant Award from the American Society for Metabolic and Bariatric Surgery (grant R03DK078562)<br><br>Protocol registration: Not reported
**Participants**	Patients with a BMI 30 kg/m2 who had undergone BS 3 years before study entry and had 50% excess WL, >21 yrs if undergone BS >=3 yrs before study enrolment and lost <50% excess weight from before surgery to study enrolment.<br><br>Exclusion criteria: BMI <30 kg/m2; participation in a weight management program in the last 6 mo; severe psychiatric disorders; pregnancy or lactation in the previous 6 months or planning to become pregnant in the next year; taking a medication known to affect the body weight in the previous 6 mo; mental retardation or psychosis; participation in a conflicting research protocol in the previous 6 mo<br><br>Number of participants screened: 230<br><br>Number of participants who met inc&exc criteria: 64<br><br>Number of patients randomized: <u>Intervention:</u> 18, <u>Control:</u> 18<br><br>Number of patients who underwent bariatric surgery: <u>Intervention:</u> 18, <u>Control:</u> 18<br><br>Type of BS: GB (n=29, 80.6%), revision of their primary surgery to a secondary GB (n=4, 11.1%), LAGB (n=2, 5.6%), and VBG (n=1, 2.8%)<br><br>Number of patients analysed at baseline: <u>Intervention:</u> 18, <u>Control:</u> 18<br><br>Age mean (SD): <u>Intervention:</u> 51.0 (7.6), <u>Control:</u> 53.9 (6.6)<br><br>Female N (%): <u>Intervention:</u> 15 (83.3%), <u>Control:</u> 17 (94.4%)<br><br>Comorbidities N (%): Not reported
**Interventions**	**Intervention:** 6-mo behavioural intervention. The patients were prescribed an exercise program according to their choice of activity. The participants were assisted in self-monitoring and setting small, incremental goals for lifestyle change. 12 weekly group meetings (lasted 1 hour) followed by 5 biweekly telephone coaching sessions (15–20 min).<br><br>**Control:** Wait list control group
**Outcomes**	Body weight and height; episodes of binge eating during the previous 6 months; current depressive symptoms
BS – bariatric surgery; COI - Conflict of interest; GB - Gastric Bypass; RCT – randomized controlled trials, SD - Standard deviation; VGB - Vertical banded gastroplasty; WL - weight loss	

Risk of bias table

Bias	Authors' judgement	Support for judgement
Random sequence generation (selection bias)	Unclear risk	Randomised, no more info
Allocation concealment (selection bias)	Unclear risk	No information
Blinding of participants and personnel (performance bias)<br>Weight loss/BMI	Low risk	Objective outcome, no blinding, but the review authors judge that the outcome was not likely to be influenced by lack of blinding
Blinding of outcome assessment (detection bias)<br>Weight loss/BMI	Low risk	Objective outcome, no blinding, but the review authors judge that the outcome was not likely to be influenced by lack of blinding
Incomplete outcome data (attrition bias)	Unclear risk	No info what type of analysis used, loss-to-follow-up similar in both groups however reasons for LTFU unreported
Selective reporting (reporting bias)	High risk	No protocol, only weight-related outcomes reported
Other bias	Low risk	None identified
BMI – body mass index; LTFU – lost to follow-up		

Kalarchian 2016

**Methods**	RCT, parallel assignment, open label, descriptive statistics, t-tests, Wilcoxon tests, chi-square analyses, Fisher's exact tests, linear regression models<br><br>Country: United States of America<br><br>Settings: Bariatric Center of Excellence at a large, urban medical center<br><br>Number of centers: 1<br><br>COI: Dr. Kalarchian reports receiving funding for research in BS from NIH/NIDDK, TOS/Nutrisystem, and ASMBS. Dr. Courcoulas reports grants from Nutrisystem, grants from EndoGastric Solutions, and other from J&J Ethicon. Drs. Cheng, Levine and Marcus have nothing to disclose<br><br>Funding: R01DK077102 from the National Institute of Diabetes and Digestive and Kidney Diseases (PI: Melissa A. Kalarchian). The sponsor had no role in the design and conduct of the study; collection, management, analysis, and interpretation of the data; or in the preparation, review, or approval of the manuscript<br><br>Protocol registration: Clinicaltrials.gov: NCT00623792
**Participants**	Inclusion Criteria: at least 18 years old, BMI > 40, or BMI 35- 40 with serious obesity-related health problems<br><br>Exclusion Criteria: mental retardation or psychosis, previously diagnosed genetic obesity syndrome, participation in a structured weight management program in the 6 mo prior to study enrolment, uncontrolled psychiatric symptomatology sufficiently severe to require immediate treatment, pregnant or lactating in the previous 6 mo, taking a medication known to affect body weight, any previous surgery for WL, deemed high risk surgical candidate.<br><br>Number of participants screened: 934<br><br>Number of participants who met inc&exc criteria: 300

	Number of patients randomized: Intervention: 121, Control: 119<br><br>Number of patients who underwent bariatric surgery: Intervention: 71, Control: 72<br><br>Type of bariatric surgery: Intervention: RYGB - 41, LAGB - 30, Control: RYGB - 46, LAGB - 26<br><br>Number of patients analysed at baseline: Intervention: 71, Control: 72<br><br>Age mean (SD): Intervention: 43.9 (10.3), Control: 45.9 (11.6)<br><br>Female N (%): Intervention: 64 (90.1%), Control: 65 (90.3%)<br><br>Comorbidities N (%): Not reported
**Interventions**	**Intervention:** 6-mo, evidence-informed, manualized behavioural lifestyle intervention, a combination of individual, face-to-face counselling sessions and telephone coaching , the first 2 months consisted of 8 weekly individual, face-to-face sessions (each 1 h). Weekly contacts continued for the next 4 months, consisting of one individual, face-to-face session and three telephone coaching sessions per month (15-20 min). Thus, the intervention consisted of a total of 12 individual, face-to-face sessions and 12 telephone contacts.<br><br>**Control:** Treatment as usual - patients received a synopsis of the information provided to participants in the lifestyle intervention
**Outcomes**	Primary Outcome Measures: weight and related outcomes (BMI, cardiovascular risk, quality of life), preparation for surgery (eating, activity and knowledge of surgery), compliance and eating problems (vomiting, dumping, plugging etc.), surgical complications and outpatient visits Secondary Outcome Measures: Weight/BMI trajectory
BMI – body mass index; COI - Conflict of interest; LAGB - Laparoscopic adjustable gastric banding; RCT – randomized controlled trials, RYGB - Roux-en-Y gastric bypass; SD - Standard deviation; WL - weight loss	

Risk of bias table

**Bias**	**Authors' judgement**	**Support for judgement**
Random sequence generation (selection bias)	Low risk	Block randomization with a block size of 4, stratified by BMI; the allocation sequence was generated by the study statistician.
Allocation concealment (selection bias)	Unclear risk	No information
Blinding of participants and personnel (performance bias) Weight loss/BMI	Low risk	Objective outcome, weight measured with a digital scale at the study office, at 6 months weight was collected from the electronic medical record for participants who were unable to complete the assessment at the study office, height measured with a mounted stadiometer - impossible to blind a patient throughout the whole study as he could guess from sessions format in which group he was placed
Blinding of participants and personnel (performance bias) Psychological outcomes	High risk	Filled out by patients
Blinding of participants and personnel (performance bias) Nonnormative eating pattern	High risk	Filled out by patients

Blinding of outcome assessment (detection bias) Weight loss/BMI	Low risk	Objective outcome, weight measured with a digital scale at the study office, at 6 months weight was collected from the electronic medical record for participants who were unable to complete the assessment at the study office, height measured with a mounted stadiometer - impossible to blind a patient throughout the whole study as he could guess from sessions format in which group he was placed
Blinding of outcome assessment (detection bias) Psychological outcomes	High risk	Filled out by patients
Blinding of outcome assessment (detection bias) Nonnormative eating pattern	High risk	Filled out by patients
Incomplete outcome data (attrition bias)	High risk	Per-protocol analysis, Serious loss of follow-up
Selective reporting (reporting bias)	High risk	Protocol available but a few outcomes not reported: total body fat, total lean mass
Other bias	Low risk	None identified
BMI – body mass index		

Lier 2012

**Methods**	RCT, parallel assignment, open label, analysis of variance, a two way contingency table analysis<br><br>Country: Norway<br><br>Settings: Department of Surgery at Haugesund Hospital on the West coast of Norway<br><br>Number of centers: 1<br><br>COI: The authors report no conflict of interest. The authors alone are responsible for the content and writing of the paper<br><br>Funding: Grant from the Western Regional Health Authority, Norway<br><br>Protocol registration: Clinicaltrials.gov: NCT00635011
**Participants**	Inclusion Criteria: being on a waiting list for BS at Haugesund hospital<br><br>Exclusion Criteria: psychosis and suicidality, suicidal attempts the last two years, or suicidal thoughts or plans.<br><br>Number of participants screened: 169

	Number of participants who met inc&exc criteria: 144<br><br>Number of patients randomized: Intervention: 49, Control: 50<br><br>Number of patients who underwent bariatric surgery: Intervention: 44, Control: 43<br><br>Type of bariatric surgery: Gastric bypass (100%)<br><br>Number of patients analysed at baseline: Intervention: 49, Control: 48<br><br>Age mean (SD): Intervention: 43.5 (11.1), Control: 42.4 (9.1)<br><br>Female N (%): Intervention: 36 (74%), Control: 32 (67%)<br><br>Comorbidities N (%): Not reported
**Interventions**	**Intervention:** Cognitive-behavioral treatment program with one preoperative group session (3h) weekly for six weeks and three postoperative group sessions (about six months, one year and two years after surgery). Homework: diary for food intake, diary for planned and executed exercise, and mindfulness training (20–30 min, six days a week).<br><br>**Control:** Treatment as usual - two 4-hours educational seminars: one preoperative and one postoperative.
**Outcomes**	Primary Outcome Measures: psychiatric comorbidity<br><br>Secondary Outcome Measures: WL
COI - Conflict of interest; RCT – randomized controlled trials; SD - Standard deviation; WL - weight loss	

Risk of bias table

**Bias**	**Authors' judgement**	**Support for judgement**
Random sequence generation (selection bias)	Low risk	Blocked randomization; block size of ten
Allocation concealment (selection bias)	Low risk	Concealed at an external research site
Blinding of participants and personnel (performance bias)<br>Weight loss/BMI	Unclear risk	No blinding, open label
Blinding of participants and personnel (performance bias)<br>Psychological outcomes	High risk	Filled out by patients, open study, outcome could be influenced
Blinding of outcome assessment (detection bias)<br>Weight loss/BMI	Unclear risk	No blinding, open label
Blinding of outcome assessment (detection bias)<br>Psychological outcomes	High risk	Filled out by patients, open study, outcome could be influenced

Incomplete outcome data (attrition bias)	High risk	Patients excluded after randomisation, 35% of patients excluded from the analysis
Selective reporting (reporting bias)	High risk	QoL missed
Other bias	Low risk	None identified
BMI – body mass index		

Paul 2021

**Methods**	RCT, parallel assignment, open label, t tests and linear mixed models<br><br>Country: Netherlands<br><br>Settings: general hospital, Rotterdam, Netherlands<br><br>Number of centers: 3<br><br>COI: No conflict of interest was reported by the authors<br><br>Funding: Not reported<br><br>Protocol registration: https://www.trialregister.nl/trial/3960
**Participants**	Patients before BS;<br><br>Inclusion criteria: successfully passing preoperative screening, being on the waiting list for bariatric surgery in one of the hospitals, age between 21 and 65 years<br><br>Exclusion criteria: being currently treated by a dietitian, psychiatrist, or psychologist; suffering from psychotic or bipolar disorder, suicidality, or substance addiction; poor command of the Dutch language; being involved in another study on weight and bariatric surgery outcomes<br><br>Number of participants screened: 213<br><br>Number of participants who met inc&exc criteria: 130<br><br>Number of patients randomized: Intervention: 65, Control: 65<br><br>Number of patients who underwent bariatric surgery: Intervention: 53, Control: 54<br><br>Type of bariatric surgery: gastric bypass (100%)<br><br>Number of patients analysed at baseline: Intervention: 65, Control: 65<br><br>Age mean (SD): Intervention: 44.1 (8.2), Control: 39.3 (10.6)<br><br>Female N (%): Intervention: 46 (73%), Control: 49 (75%)

	Comorbidities N (%): not reported
**Interventions**	**Intervention:** cognitive behavioural therapy of 10 individual sessions of 45 minutes, conducted by a psychologist or cognitive behavioural therapeutic worker.<br><br>**Control:** conventional preperation procedure consisting of an information meeting by the surgeon or nurse practitioner and an information meeting by the dietitian. Patients also receive a detailed patient information booklet.
**Outcomes**	Weight, eating behaviour, eating disorders, depression, quality of life, psychological distress
COI - Conflict of interest; EBW - excessive body weight; RCT – randomized controlled trials, SD - Standard deviation	

Risk of bias table

**Bias**	**Authors' judgement**	**Support for judgement**
Random sequence generation (selection bias)	Low risk	An online randomization list (www.randomization.com)
Allocation concealment (selection bias)	Low risk	An independent PsyQ office manager manager the randomization process
Blinding of participants and personnel (performance bias)<br>Weight loss/BMI	Unclear risk	Objective outcome, open study, outcome could be influenced
Blinding of participants and personnel (performance bias)<br>Psychological outcomes	High risk	Open study, outcome could be influenced
Blinding of participants and personnel (performance bias)<br>Quality of life	High risk	Open study, outcome could be influenced
Blinding of participants and personnel (performance bias)<br>Nonnormative eating pattern	High risk	Open study, outcome could be influenced
Blinding of outcome assessment (detection bias)<br>Weight loss/BMI	Unclear risk	Objective outcome, open study, outcome could be influenced
Blinding of outcome assessment (detection bias)<br>Psychological outcomes	High risk	Open study, outcome could be influenced
Blinding of outcome assessment (detection bias)<br>Quality of life	High risk	Open study, outcome could be influenced

Blinding of outcome assessment (detection bias)<br><br>Nonnormative eating pattern	High risk	Open study, outcome could be influenced
Incomplete outcome data (attrition bias)	Unclear risk	For all outcomes missing data reasons reported, ITT analysis, but no info about imputations, small and balanced number of missing patients
Selective reporting (reporting bias)	Unclear risk	Differences in the description of weight outcome between a protocol and a manuscript
Other bias	Low risk	None identified
BMI – body mass index, ITT – intention-to-treat		

Petasne 2013

**Methods**	RCT, parallel assignment, open label, t tests and regression analysis<br><br>Country: United States of America<br><br>Settings: Laparoscopic Institute of South Florida<br><br>Number of centers: 1<br><br>COI: No conflict of interest was reported by the authors<br><br>Funding: Not reported<br><br>Protocol registration: Not reported
**Participants**	Patients after BS, who completed Phase 1;<br><br>Inclusion criteria: having undergone a LRYGB 24 2 weeks before recruitment and being Hispanic American with bilingual (Spanish and English) proficiency.<br><br>Exclusion criteria: physical inability to participate, medical conditions, treatment with antidepressant medication, and/or pregnancy<br><br>Number of participants screened: 307<br><br>Number of participants who met inc&exc criteria: 292<br><br>Number of patients randomized: Intervention: 72, Control: 72<br><br>Number of patients who underwent bariatric surgery: Intervention: 72, Control: 72<br><br>Type of bariatric surgery: LRYGB (100%)<br><br>Number of patients analysed at baseline: Intervention: 72, Control: 72<br><br>Age mean (SD): Intervention: 44.2 (12.6), Control: 44.8 (14.4)

	Female N (%): Intervention: 62 (86.1%), Control: 58 (80.6%)<br><br>Comorbidities N (%):<br><br>Smoking: Intervention: 10 (13.9%), Control: 14 (19.4%)
**Interventions**	**Intervention:** Comprehensive nutrition and lifestyle educational intervention: 6 nutrition and lifestyle education and behavioural-motivational sessions in groups of up to 12 participants in Spanish or English according to the participant's language preference.<br><br>**Control:** Brief printed guidelines for healthy eating and physical activity at the first assessment.
**Outcomes**	Change in depression scores, EBW loss, attendance at the comprehensive educative sessions; visits to psychologists
COI - Conflict of interest; EBW - excessive body weight; LRYGB - Laparoscopic Roux-en-Y gastric bypass; RCT – randomized controlled trials, SD - Standard deviation	

Risk of bias table

**Bias**	**Authors' judgement**	**Support for judgement**
Random sequence generation (selection bias)	Unclear risk	Not reported
Allocation concealment (selection bias)	Unclear risk	No details reported
Blinding of participants and personnel (performance bias)<br>Weight loss/BMI	Unclear risk	Objective outcome, open study, outcome could be influenced
Blinding of participants and personnel (performance bias)<br>Psychological outcomes	High risk	Open study, outcome could be influenced
Blinding of outcome assessment (detection bias)<br>Weight loss/BMI	Unclear risk	Objective outcome, open study, outcome could be influenced
Blinding of outcome assessment (detection bias)<br>Psychological outcomes	High risk	Open study, outcome could be influenced
Incomplete outcome data (attrition bias)	Unclear risk	For all outcomes missing data reasons reported, ITT analysis, but no info about imputations, small and balanced number of missing patients
Selective reporting (reporting bias)	Unclear risk	The study protocol is not available
Other bias	Low risk	None identified

BMI – body mass index, ITT – intention-to-treat

Tucker 1991

**Methods**	RCT, parallel assignment, open label, ANOVA, ANCOVA, t test<br><br>Country: United States of America<br><br>Settings: Not reported<br><br>Number of centers: Not reported<br><br>COI: Not reported<br><br>Funding: Not reported<br><br>Protocol registration: Not reported
**Participants**	Inclusion criteria: The positive psychological benefits of behaviour therapy<br><br>Number of participants screened: 60<br><br>Number of participants who met inc&exc criteria: 50<br><br>Number of patients randomized: Both groups altogether: 41<br><br>Number of patients who underwent bariatric surgery: Intervention: 17, Control: 15<br><br>Type of bariatric surgery: Both groups altogether: GB - 10, VBG - 22<br><br>Number of patients analysed at baseline: Intervention: 17, Control: 15<br><br>Age mean: Both groups altogether: 40.18<br><br>Female N (%): Both groups altogether: 21 (65.6)<br><br>Comorbidities N (%): Not reported
**Interventions**	The 12 sets of written materials mailed every 2 weeks to treatment subjects (behavioural approach to weight management; nutritional considerations, modification of eating topography, behavioural problem-solving strategies
**Outcomes**	Weight and blood pressure which were measured at each visit.
COI - Conflict of interest; GB - Gastric Bypass; RCT – randomized controlled trials; VGB - Vertical banded gastroplasty;	

Risk of bias table

Bias	Authors' judgement	Support for judgement
Random sequence generation (selection bias)	Unclear risk	Not reported
Allocation concealment (selection bias)	Unclear risk	No details reported
Blinding of participants and personnel (performance bias)<br>Weight loss/BMI	Unclear risk	Objective outcome, open study, outcome could be influenced
Blinding of outcome assessment (detection bias)<br>Weight loss/BMI	Unclear risk	Objective outcome, open study, outcome could be influenced
Incomplete outcome data (attrition bias)	High risk	Per-protocol analysis, outcome data and analysis not clearly described, it seems that 60 pts were randomised and only half of them were included into the analysis
Selective reporting (reporting bias)	Unclear risk	The study protocol is not available
Other bias	Low risk	None identified
BMI – body mass index		

Wild 2017

**Methods**	Randomized controlled two-armed multi-centre trial, Pearson's correlation coefficients, sensitivity analysis<br><br>Country: Germany<br><br>Settings: University Hospital of Heidelberg, University Hospital of Tübingen, and the Hospital Sachsenhausen, Frankfurt<br><br>Number of centers: 3<br><br>COI: All authors declare no conflict of interest<br><br>Funding: Funded be the Federal Ministry of Education and Research (No. 01G10843) The funding organization had no influence on the conduct of the study, data analysis, and preparation of the manuscript<br><br>Protocol registration: www.controlled-trials.com: ISRCTN11076390
**Participants**	BMI ≥ 40kg/m² respectively BMI ≥ 35 kg/m² with co-morbidities<br><br>Number of participants screened: 306<br><br>Number of participants who met inc&exc criteria: 117

	Number of patients randomized: Intervention: 59, Control: 58<br><br>Number of patients who underwent bariatric surgery: Intervention: 57, Control: 54<br><br>Type of bariatric surgery: Intervention: LSG – 33, RYGB – 22, LAGB – 2, missing – 1, Control: LSG - 33, RYGB – 20, LAGB – 1, missing - 2<br><br>Number of patients analysed at baseline: Intervention: 58, Control: 56<br><br>Age mean (SD): Intervention: 41.2 (9.0), Control: 41.9 (9.6)<br><br>Female N (%): Intervention: 35 (60.3%), Control: 45 (80.4%)<br><br>Comorbidities N (%):<br><br>Depression: Intervention: 29 (50%), Control: 20 (38%)
**Interventions**	**Intervention:** Same as in control group + Videoconferencing-based psychoeducational group intervention: one year group program that included face-to-face and videoconferencing sessions –<br><br>**Control:** Conventional surgical visits as implemented in the ongoing clinical routine
**Outcomes**	Weight, BMI, %EWL, %TWL, quality of life, depression severity, self-efficacy, eating disorder assessment
BMI - body mass index; COI, conflict of interest; EWL – excess weight loss; LAGB - Laparoscopic adjustable gastric banding; LSG - Laparoscopic sleeve gastrectomy; RYGB - Roux-en-Y gastric bypass; SD, Standard deviation; TWL - total weight loss	

Risk of bias table

**Bias**	**Authors' judgement**	**Support for judgement**
Random sequence generation (selection bias)	Low risk	By randomization software “RANDI 2”
Allocation concealment (selection bias)	Low risk	Randomization program was applied by an independent assistant
Blinding of participants and personnel (performance bias)<br>Weight loss/BMI	Unclear risk	Objective outcome, open study, outcome could be influenced
Blinding of participants and personnel (performance bias)<br>Quality of life	High risk	Open study, outcome could be influenced
Blinding of participants and personnel (performance bias)<br>Psychological outcomes	High risk	Open study, outcome could be influenced
Blinding of participants and personnel (performance bias)<br>Self efficacy	High risk	Open study, outcome could be influenced
Blinding of participants and personnel (performance bias)<br>Nonnormative eating pattern	High risk	Open study, outcome could be influenced

Blinding of outcome assessment (detection bias)<br>Weight loss/BMI	Unclear risk	In majority reported by patients
Blinding of outcome assessment (detection bias)<br>Quality of life	High risk	Reported by patients
Blinding of outcome assessment (detection bias)<br>Psychological outcomes	High risk	Reported by patients
Blinding of outcome assessment (detection bias)<br>Self efficacy	High risk	Reported by patients
Blinding of outcome assessment (detection bias)<br>Nonnormative eating pattern	High risk	Reported by patients
Incomplete outcome data (attrition bias)	Low risk	ITT and PP analyses were performed and missing data imputed MMRM algorithm
Selective reporting (reporting bias)	Low risk	Outcomes reported as defined in protocol.
Other bias	Low risk	None identified
BMI – body mass index		

Supplementary Materials, Table S5: List of excluded studies

Study ID	Reason for exclusion
Baruch 2008	Wrong population
Bond 2015	Wrong outcomes
Bracaglia 2011	Wrong study design
Brown 2009	Wrong population
Case 2011	Wrong population
Cassin 2016	Wrong outcomes
Centro 2015	Wrong population
Centro 2017	Wrong population
Chacko 2016	Wrong population
Chacko b 2016	Wrong population
Columbia 2016	Wrong intervention
Cowan 2001	Wrong outcomes
Ana 2004	Wrong population
David 2016	Wrong outcomes
Delparte 2018	Wrong study design
Drexel 2005	Wrong population
Drexel 2008	Wrong population
Drexel 2011	Wrong population
Drexel 2012	Wrong population
Drexel 2016	Wrong study design
Duke 2007	Wrong population
Duke 2015	Wrong population
Duke 2017	Wrong population
Elakkary 2004	Wrong study design
Eli 2009	Wrong population
Federal 2015	Wrong population
Federal 2016	Wrong study design
Felix 2018	Wrong outcomes
Francine 2013	Wrong population
Garcia-Delgado 2021	Wrong intervention
Gradaschi 2019	Wrong study design
Grilo 2021	Wrong population
Grupo 2012	Wrong study design
Horber 2021	Wrong intervention
Hospices 2009	Wrong population
Hôpital 2015	Wrong population
Icahn 2013	Wrong population
Imperial 2011	Wrong population
Kaiser 2004	Wrong population
Kansas 2012	Wrong population
Lent 2019	Wrong outcomes
Linkoeping 2015	Wrong population
Marcon 2016	Wrong outcomes

Study ID	Reason for exclusion
Marcon 2017	Wrong outcomes
Matteo 2007	Wrong population
Mayo 2010	Wrong study design
Mensorio 2016	Wrong study design
National 2002	Wrong population
New Study	Wrong study design
New Studyb	Wrong study design
Norwegian 2009	Wrong study design
Novindiet 2015	Wrong population
Ochner 2007	Wrong study design
Oklahoma 2016	Wrong population
Papalazarou 2010	Wrong intervention
Parikh 2012	Wrong intervention
Qatar 2013	Wrong population
Quilez-Orden 2021	Wrong study design
Research 2008	Wrong population
Rush University Medical Center 2019	Wrong outcomes
Saunders 2004	Wrong study design
Schweiger 2015	Wrong study design
Sellberg 2018	Wrong population
Sellberg 2019	Wrong population
Sockalingam 2017	Wrong study design
Sockalingam 2019	Wrong study design
Stahre 2008	Wrong population
Stanford 2006	Wrong population
Süd 2012	Wrong population
The 2015	Wrong population
University 2011	Wrong population
University 2011a	Wrong population
University 2012	Wrong study design
University 2014	Wrong study design
Washington 1994	Wrong population
Washington 1997	Wrong population
Weineland 2011	Wrong outcomes
Weineland 2012	Wrong population
Weineland 2012a	Wrong population
Yale 2002	Wrong population
Yale 2007	Wrong population
Yale 2008	Wrong population
Yale 2017	Wrong population
Yale 2017a	Wrong population
Yale 2017b	Wrong population

Excluded studies

Baruch 2008
Baruch College City University of New York, National Institute of Diabetes, Digestive, Kidney Diseases. Managing Obesity by Combining Behavioral Weight Loss and Commercial Approaches. 2008.

Bond 2015
Bond Ds, Vithiananthan S, Thomas Jg, Trautvetter J, UnickJl, Jakicic Jm, et al. Bari-Active: a randomized controlled trial of a preoperative intervention to increase physical activity in bariatric surgery patients. 2015;11(1):169-77.

Bracaglia 2011
Bracaglia R, D'Ettorre M, Gniuli D, Gigliofiorito P, Gentileschi S, Mingrone G. Morbidly obese patients undergoing bariatric and body contouring surgery: Psychological evaluation after treatments. Journal of Plastic, Reconstructive and Aesthetic Surgery 2011;64(9):1246-8.

Brown 2009
Brown University, National Institute of Mental Health. Integrated Treatment for Comorbid Depression and Obesity in Adolescents. 2009.

Case 2011
Case Western Reserve University, National Heart Lung, Blood Institute. Ideas Moving Parents and Adolescents to Change Together (IMPACT). 2011.

Cassin 2016
Cassin Se, Sockalingam S, Du C, Wnuk S, Hawa R, Parikh Sv. A pilot randomized controlled trial of telephone-based cognitive behavioural therapy for preoperative bariatric surgery patients. 2016;80:17-22.

Centro 2015
Centro Hospitalar Lisboa Norte, Faculdade de Medicina de Lisboa, Faculdade de Psicologia e de Ciências de Educação da Universidade do Porto. Efficacy of Motivational Interview in the Treatment of Obesity and Overweight in Adolescents. 2015.

Centro 2017
Centro Mente Aberta de Mindfulness, Conselho Nacional de Desenvolvimento Científico e Tecnológico. Mindfulness Intervention for Overweight Primary Care Patients. 2017.

Chacko 2016
Beth Israel Deaconess Medical Center, Center for Nutritional Research Charitable Trust. A Trial of Lifestyle Interventions to Control Weight After Bariatric Surgery. https://ClinicalTrials.gov/show/NCT02603601 2014.

Chacko b 2016
Chacko SA, Yeh GY, Davis RB, Wee CC. A mindfulness-based intervention to control weight after bariatric surgery: Preliminary results from a randomized controlled pilot trial. Complementary therapies in medicine 2016;28:13-21.

Columbia 2016
Columbia University, National Institute of Diabetes, Digestive, Kidney Diseases. Mechanisms Underlying Predictors of Success From Obesity Surgery. 2016.

Cowan 2001
Cowan Gs, Buffington Ck, Cowan Gs, Hathaway D. Assessment of the effects of a taped cognitive behavior message on postoperative complications (therapeutic suggestions under anesthesia). 2001;11(5):589-93.

Damaso 2004
Damaso AR, da Silveira Campos RM, Castro SE, Pelissari Kravchychyn AC. The Effect of 1 Year of Multiprofessional Therapy on Obesity. 2004.

David 2016
David LA, Sockalingam S, Wnuk S, Cassin SE. A pilot randomized controlled trial examining the feasibility, acceptability, and efficacy of Adapted Motivational Interviewing for post-operative bariatric surgery patients. Eating behaviors 2016;22:87-92.

Delparte 2018
Delparte CA, Power HA, Gelinas BL, Oliver AM, Hart RD, Wright KD. Examination of the effectiveness of a brief, adapted dialectical behavior therapy- skills training group for bariatric surgical candidates. Obesity Surgery 2019;29(1):252-61.

Drexel 2005
Drexel University, National Institute of Diabetes, Digestive, Kidney Diseases. Weight Loss Maintenance in Primary Care. 2005.

Drexel 2008
Drexel University, National Institute of Diabetes, Digestive, Kidney Diseases. Behavioral Treatment for Weight Loss. 2008.

Drexel 2011
Drexel University. Environmental and Acceptance-Based Innovations for Weight Loss Maintenance. 2011.

Drexel 2012
Drexel University. Acceptance-based Behavioral Treatment for Obesity: Maintenance and Mechanisms. 2012.

Drexel 2016
Drexel University. Project BITE: Binge Intervention Target Effectiveness. 2016.

Duke 2007
Duke University, University of Pennsylvania, National Center for Complementary, Integrative Health. Engaging Motivation for the Prevention of Weight Regain. 2007.

Duke 2015
Duke University, University of North Carolina Chapel Hill. Integrated Child Obesity Treatment Study: Bull City Healthy and Fit. 2015.

Duke 2017
Duke University. Charge: A Text Messaging-based Weight Loss Intervention. 2017.

Elakkary 2004
Elakkary E, Gazayerli MM. Laparoscopic adjustable gastric band: Do support groups add to the weight loss? OBES. SURG. 2004;14(8):1139-40.

Eli 2009
Eli Lilly, Company. A Weight Loss Study in Overweight Men and Women. 2009.

Federal 2015
Federal University of São Paulo, University of Western Sydney. Healthy approach to weight management and Food in Eating Disorders (HAPIFED). 2015.

Federal 2016
Federal University of São Paulo, Conselho Nacional de Desenvolvimento Científico e Tecnológico, Fundação de Amparo à Pesquisa do Estado de São Paulo, Coordination for the Improvement of Higher Education Personnel. Interdisciplinary Intervention in Patients With Obesity and Other Metabolic Diseases. 2016.

Felix 2018
Felix M, Ferreira MBG, Oliveira LF, Barichello E, Pires PDS, Barbosa MH. Guided imagery relaxation therapy on preoperative anxiety: a randomized clinical trial. Revistalatino-americana de enfermagem. 2018;26:e3101.

Francine 2013
Francine Rosselli, Wesleyan University. Binge Eating Self-help Treatment for University Students. https://ClinicalTrials.gov/show/NCT01888406 2013.

Garcia-Delgado 2021

Garcia-Delgado, Y., Lopez-Madrazo-hernandez, M., Alvarado-Martel, D., Miranda-Calderin, G., Ugarte-Lopetegui, A., Gonzalez-Medina, R., . . . et al. (2021). Prehabilitation for bariatric surgery: a randomized, controlled trial protocol and pilot study. 13(9).

Gradaschi 2019
Gradaschi R, Molinari V, Sukkar SG, De Negri P, Adami GF, Camerini G. Effects of the Postoepartive Dietetic/BehavioralCounseling on the Weight Loss After Bariatric Surgery. Obesity surgery. 2019.

Grilo 2021

Grilo, C., Ivezaj, V., Duffy, A., & Gueorguieva, R. (2021). Randomized Controlled Trial of Treatments for Loss-of-Control Eating Following Bariatric Surgery. 29(4), 689-697.

Grupo 2012
GrupoLusófona, Fundação para a Ciência e a Tecnologia, University of Lisbon. Treatment of Pediatric Obesity (TOP) - A Multidisciplinary Approach Involving Adolescents and Their Peers. https://ClinicalTrials.gov/show/NCT02024061 2012.

Hôpital 2015
Hôpital le Vinatier. Early and Short Psycho-educational Lifestyle Intervention. https://ClinicalTrials.gov/show/NCT02657941 2015.

Horber 2021

Horber, F. F., & Steffen, R. (2021). Reversal of Long-Term Weight Regain After Roux-en-Y Gastric Bypass Using Liraglutide or Surgical Revision. A Prospective Study. Obesity surgery, 31(1), 93-100.

Hospices 2009
Hospices Civils de Lyon. Sleep and Obesity in Teenagers. https://ClinicalTrials.gov/show/NCT00841347 2009.

Icahn 2013
Icahn School of Medicine at Mount Sinai, NoomInc, Kaiser Foundation Hospitals Center for Health Research, National Institute of Mental Health. Smartphone Technology and CBT-GSH in Binge Eaters. https://ClinicalTrials.gov/show/NCT02399982 2013.

Imperial 2011
Imperial College London, National Institute for Health Research United Kingdom. Bariatric Surgery and Guided Self-help for Binge Eating Disorder. https://ClinicalTrials.gov/show/NCT02094027 2011.

Kaiser 2004
Kaiser Permanente, National Institute of Mental Health. Guided Self-Help Treatment for Binge Eating Disorder. https://ClinicalTrials.gov/show/NCT00158340 2004.

Kansas 2012
Kansas State University. The Wildcat Wellness Coaching Trial. https://ClinicalTrials.gov/show/NCT01845480 2012.

Lent 2019
Lent MR, Campbell LK, Kelly MC, Lawson JL, Murakami JM, Gorrell S, et al. The feasibility of a behavioral group intervention after weight-loss surgery: A randomized pilot trial. PLoS ONE 2019;14(10).

Linkoeping 2015
Linkoeping University. Individually Tailored Web-based Cbt for Eating Disorders and the Role of Knowledge Acquisition. https://ClinicalTrials.gov/show/NCT02700620 2015.

Marcon 2016
Marcon Er, Baglioni S, Bittencourt L, Lopes Cln, Neumann Cr, TrindadeMrm. What Is the Best Treatment before Bariatric Surgery? Exercise, Exercise and Group Therapy, or Conventional Waiting: a Randomized Controlled Trial. 2016;1-11.

Marcon 2017
MarconEr, Baglioni S, Bittencourt L, Lopes Cln, Neumann Cr, TrindadeMrm. What Is the Best Treatment before Bariatric Surgery? Exercise, Exercise and Group Therapy, or Conventional Waiting: a Randomized Controlled Trial. 2017;27(3):763-73.

Matteo 2007
Matteo IrccsPoliclinico S. Effectiveness of Cognitive Behavioral Therapy vs. Prescriptive Diet in Short and Medium-term Control of Body Weight. https://ClinicalTrials.gov/show/NCT01686854 2007.

Mayo 2010
Mayo Clinic. A Psychological and Behavioral Intervention for Post-Bariatric Patients. https://ClinicalTrials.gov/show/NCT01453517 2010.

Mensorio 2016
Mensorio M, Costa-Júnior Á. Psychological intervention to candidates for surgery bariatric in a public hospital in Brazil. Revista Peruana de Medicina Experimental y SaludPublica 2016;33(1):120-7.

National 2002
National Institute of Diabetes, Digestive, Kidney Diseases, Hoffmann-La Roche. Effectiveness of Brief Counseling for Weight Management. https://ClinicalTrials.gov/show/NCT00212199 2002.

New Study
Identifying, evaluating, and treating overweight and obesity in adults. Revista Panamericana de Salud Publica/Pan American Journal of Public Health 2001;10(2):118-24.

New Studyb
64th Scientific Sessions of the American Diabetes Association. Practical Diabetes International 2004;21(8):314-316a.

Norwegian 2009
Norwegian School of Sport Sciences, Hjelp. Lifestyle intervention for the Treatment of Severe Obesity. https://ClinicalTrials.gov/show/NCT01675713 2009.

Novindiet 2015
Novindiet Clinic, Tehran University of Medical Sciences, University of Nottingham. Effects of Cognitive Behavioral Therapy (CBT) on Weight Maintenance After Successful Weight Loss. https://ClinicalTrials.gov/show/NCT02398253 2015.

Ochner 2007
Ochner C N, Geliebter A. Binge eating disorder. Management 2007;3(4):161-4.

Oklahoma 2016
Oklahoma State University, Kent State University, Case Western Reserve University, University of Minnesota Clinical, Translational Science Institute, Drexel University, et al. Cognitive and Self-regulatory Mechanisms Of Obesity Study. https://ClinicalTrials.gov/show/NCT02786238 2016.

Papalazarou 2010
Papalazarou A, Yannakoulia M, Kavouras SA, Komesidou V, Dimitriadis G, Papakonstantinou A, et al.. Lifestyle Intervention Favorably Affects Weight Loss and Maintenance Following Obesity Surgery. Obesity (Silver Spring) 2010;18(7):1348-53..

Parikh 2012
Parikh M, Dasari M, McMacken M, Ren C, Fielding G, Ogedegbe G. Does a preoperative medically supervised weight loss program improve bariatric surgery outcomes? A pilot randomized study. 2012;26(3):853-61.

Qatar 2013
Qatar University, Imperial College London, Hamad Medical Corporation, Supreme Council Of Health Qatar, Aspetar, Leeds Beckett University. Adapted Cognitive Behavioral Approach to Addressing Overweight and Obesity Among Qatari Youth. https://ClinicalTrials.gov/show/NCT02972164 2013.

Quilez-Orden 2021

Quilez-Orden, A., Ferreres-Galan, V., & Osma, J. (2020). Feasibility and Clinical Usefulness of the Unified Protocol in Online Group Format for Bariatric Surgery Candidates: Study Protocol for a Multiple Baseline Experimental Design. International journal of environmental research and public health, 17(17).

Research 2008
Research V A Office of, Development. Cognitive Behavioral Therapy for Obesity. https://ClinicalTrials.gov/show/NCT00569517 2008.

Rush University Medical Center 2019
Rush University Medical Center. Post-Bariatric Weight Regain Behavioral Intervention. https://ClinicalTrials.gov/show/NCT03564392.

Saunders 2004
Saunders R. Post-surgery group therapy for gastric bypass patients. Obesity surgery 2004;14(8):1128-31.

Schweiger 2015
Schweiger U. Counseling intervention helps little in obesity. MMW Fortschritte der Medizin 2015;157(9):39.

Sellberg 2018
SellbergF, Possmark S, Ghaderi A, Näslund E, Willmer M, Tynelius P, et al. A dissonance-based intervention for women post roux-en-Y gastric bypass surgery aiming at improving quality of life and physical activity 24 months after surgery: study protocol for a randomized controlled trial. BMC Surgery 2018; 18(1):25.

Sellberg 2019
Sellberg F, Possmark S, Willmer M, Tynelius P, Berglind D. One-year follow-up of a dissonance-based intervention on quality of life, wellbeing, and physical activity after Roux-en-Y gastric bypass surgery: a randomized controlled trial. Surgery for Obesity and Related Diseases. 2019

Sockalingam 2017
Sockalingam S, Cassin S E, Wnuk S, Du C, Jackson T, Hawa R, et al. A Pilot Study on Telephone Cognitive Behavioral Therapy for Patients Six-Months Post-Bariatric Surgery. Obesity Surgery 2017;27(3):670-5.

Sockalingam 2019
Sockalingam S, Cassin S E, Wnuk S, Du C, Jackson T, Hawa R, et al. A Pilot Study on Telephone Cognitive Behavioral Therapy for Patients Six-Months Post-Bariatric Surgery. Obesity Research and Clinical Practice 2019;13(5): 499-504.

Stahre 2008
Stahre L. Lakartidningen 2008;105(38):2572-4.

Stanford 2006
Stanford University, American Heart Association. Behavioral Strategies for Weight Management. https://ClinicalTrials.gov/show/NCT00575731 2006.

Süd 2012
Süd F E M. Web Based Follow up Intervention in Obesity Treatment for Women. https://ClinicalTrials.gov/show/NCT01675687 2012.

The 2015
The Miriam Hospital, National Institute of Diabetes, Digestive, Kidney Diseases. Using Novel Behavioral Approaches to Improve Long-Term Weight Loss Outcomes. https://ClinicalTrials.gov/show/NCT02156752 2015.

University 2011
University of North Carolina Chapel Hill, National Institute of Diabetes, Digestive, Kidney Diseases. Family PALS (PArtners in Lifestyle Support). https://ClinicalTrials.gov/show/NCT01324011 2011.

University 2011a
University of Wuerzburg. Severe Obesity: Bariatric Surgery vs. Life-Style-Intervention. https://ClinicalTrials.gov/show/NCT01352403 2011.

University 2012
University Health Network Toronto. Telephone-based Cognitive Behavioral Therapy for Bariatric Surgery Patients: A Pilot Study. https://ClinicalTrials.gov/show/NCT01508585 2012.

University 2014
University Health Network Toronto. Tele-CBT One Year Following Bariatric Surgery: A Pilot Study. https://ClinicalTrials.gov/show/NCT02920112 2014.

Washington 1994
Washington University School of Medicine. Psychological Treatment of Overweight Binge Eaters. https://ClinicalTrials.gov/show/NCT01208272 1994.

Washington 1997
Washington University School of Medicine. The Psychological Treatment of Overweight Binge Eaters Minority Supplement. https://ClinicalTrials.gov/show/NCT01208259 1997.

Weineland 2011
Weineland Sm, Arvidsson D, Kakoulidis T, Dahl J. Acceptance and commitment therapy for bariatric surgery patients, a pilot rct. 2011;21(8):1044.

Weineland 2012
Weineland S, Hayes S C, Dahl J. Psychological flexibility and the gains of acceptance-based treatment for post-bariatric surgery: Six-month follow-up and a test of the underlying model. Clinical 2012;2(1-2):15-24.

Weineland 2012a
Weineland S, Arvidsson D, KakoulidisTp, Dahl J. Acceptance and commitment therapy for bariatric surgery patients, a pilot RCT. Obesity Research & Clinical Practice 2012;6(1):e21-30.

Yale 2002
Yale University, National Institute of Diabetes, Digestive, Kidney Diseases. Treatment for Obesity and Binge Eating Disorder. https://ClinicalTrials.gov/show/NCT00537758 2002.

Yale 2007
Yale University, National Institutes of Health, National Institute of Diabetes, Digestive, Kidney Diseases. Treatment of Binge Eating in Obese Patients in Primary Care. https://ClinicalTrials.gov/show/NCT00537810 2007.

Yale 2008
Yale University, National Institute of Diabetes, Digestive, Kidney Diseases. Treatment of Obesity and Binge Eating: Behavioral Weight Loss Versus Stepped Care. https://ClinicalTrials.gov/show/NCT00829283 2008.

Yale 2017
Yale University, National Institute of Diabetes, Digestive, Kidney Diseases. Behavioral and Pharmacologic Treatment of Binge Eating and Obesity: Specialist Treatment. https://ClinicalTrials.gov/show/NCT03063606 2017.

Yale 2017a
Yale University, National Institute of Diabetes, Digestive, Kidney Diseases. Behavioral and Pharmacologic Treatment of Binge Eating and Obesity: Acute Treatment. https://ClinicalTrials.gov/show/NCT03045341 2017.

Yale 2017b
Yale University, National Institute of Diabetes, Digestive, Kidney Diseases. Behavioral and Pharmacologic Treatment of Binge Eating and Obesity: Maintenance Therapy. https://ClinicalTrials.gov/show/NCT03047005 2017.

Supplementary Materials, Figure S1: Graphical presentation of review authors' judgements about each risk of bias item for each included study.

	Random sequence generation (selection bias)	Allocation concealment (selection bias)	Blinding of participants and personnel (performance bias): Weight loss/BMI	Blinding of participants and personnel (performance bias): Quality of life	Blinding of participants and personnel (performance bias): Psychological outcomes	Blinding of participants and personnel (performance bias): Nonnormative eating pattern	Blinding of outcome assessment (detection bias): Weight loss/BMI	Blinding of outcome assessment (detection bias): Quality of life	Blinding of outcome assessment (detection bias): Psychological outcomes	Blinding of outcome assessment (detection bias): Nonnormative eating pattern	Incomplete outcome data (attrition bias)	Selective reporting (reporting bias)	Other bias
Hjelmesæth 2018	+	+	+		−	−	+		−	−	−	+	+
Hollywood 2015	?	+	?	−	−	−	?	−	−	−	−	+	+
Kalarchian 2012	?	?	+				+				?	−	+
Kalarchian 2016	+	?	+		−	−	+		−	−	−	−	+
Lier 2012	+	+	?		−		?		−		−	−	+
Paul 2021	+	+	?	−	−	−	?	−	−	−	?	?	+
Petasne 2013	?	?	?		−		?		−		?	?	+
Tucker 1991	?	?	?				?				−	?	+
Wild 2017	+	+	?	−	−	−	?	−	−	−	+	+	+

Supplementary Materials, Figure S2: Review authors' judgements about each risk of bias item presented as percentages across all included studies.

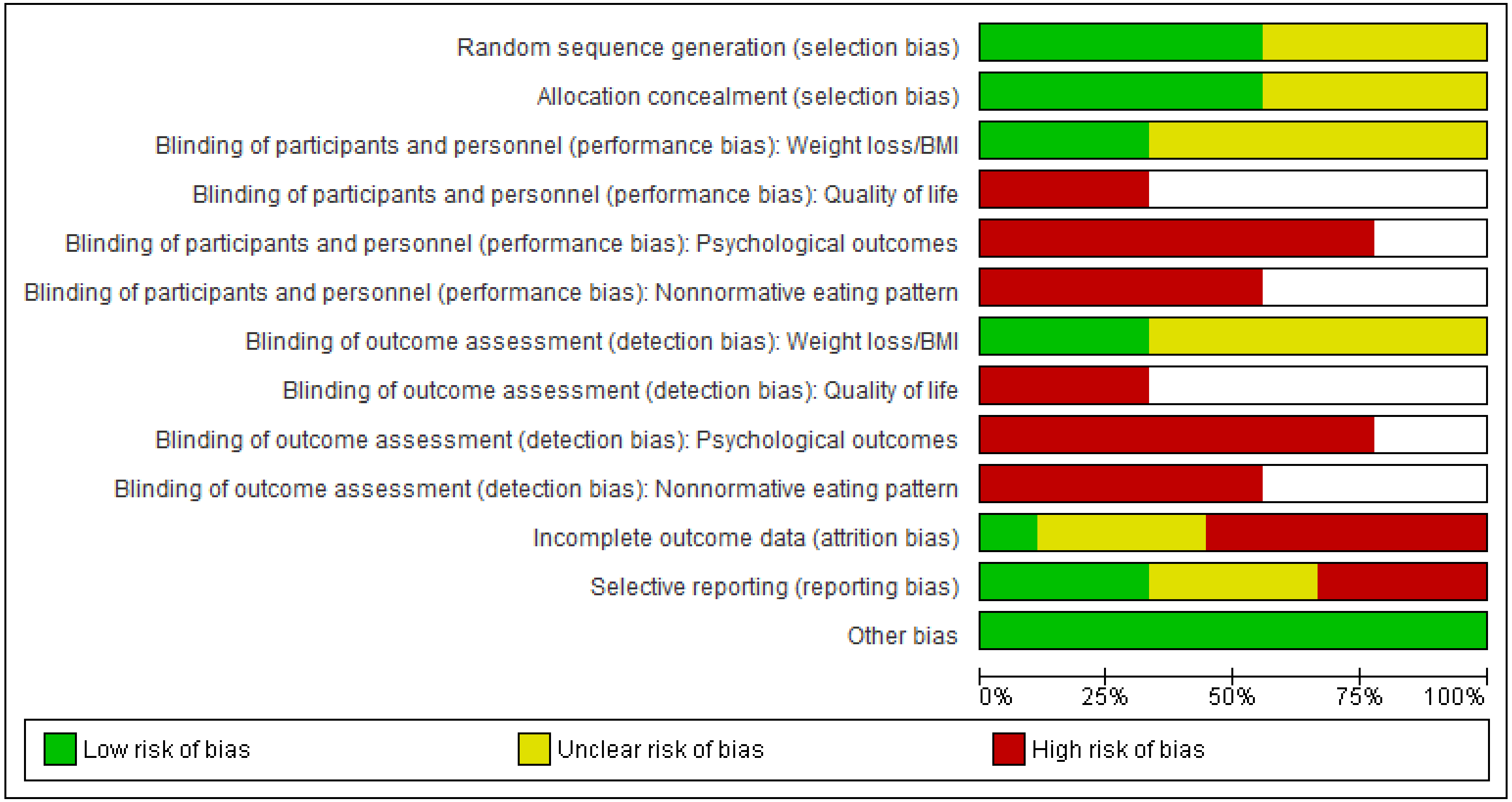

BMI: body mass index

Supplementary Materials, Figure S3: Comparison between psychological intervention versus any control in the change of BMI at 6-12 months follow-up

Posterior Estimates per Model

				95% Credible Interval		
		Mean	SD	Lower	Upper	BF_{01}
Random effects	μ	−0.287	0.643	−1.689	0.909	1.540
	τ	0.354	0.506	0.040	1.737	1.129[a]

Note. μ and τ are the group-level effect size and standard deviation, respectively.
[a] Bayes factor of the fixed effects H_1 over the random effects H_1.

Forest Plot

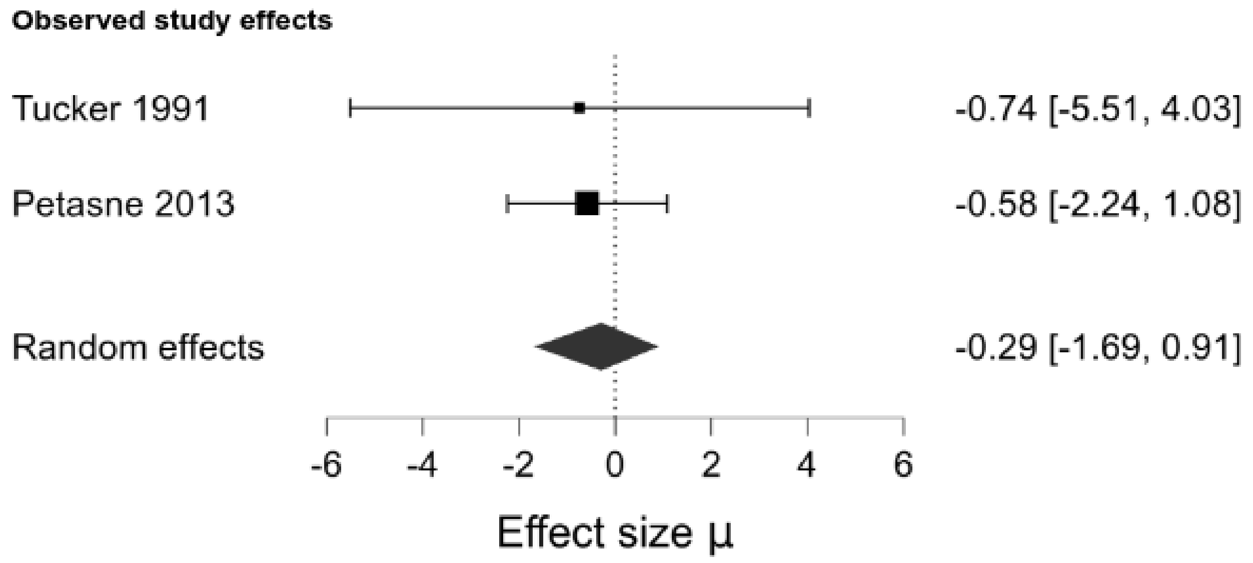

Supplementary Materials, Figure S4: Comparison between psychological intervention versus any control in the change of BMI at 1-2 years follow-up

Posterior Estimates per Model

				95% Credible Interval		
		Mean	SD	Lower	Upper	BF_{01}
Random effects	μ	−0.588	0.371	−1.338	0.120	0.590
	τ	0.358	0.376	0.041	1.366	0.988[a]

Note. μ and τ are the group-level effect size and standard deviation, respectively.
[a] Bayes factor of the fixed effects H_1 over the random effects H_1.

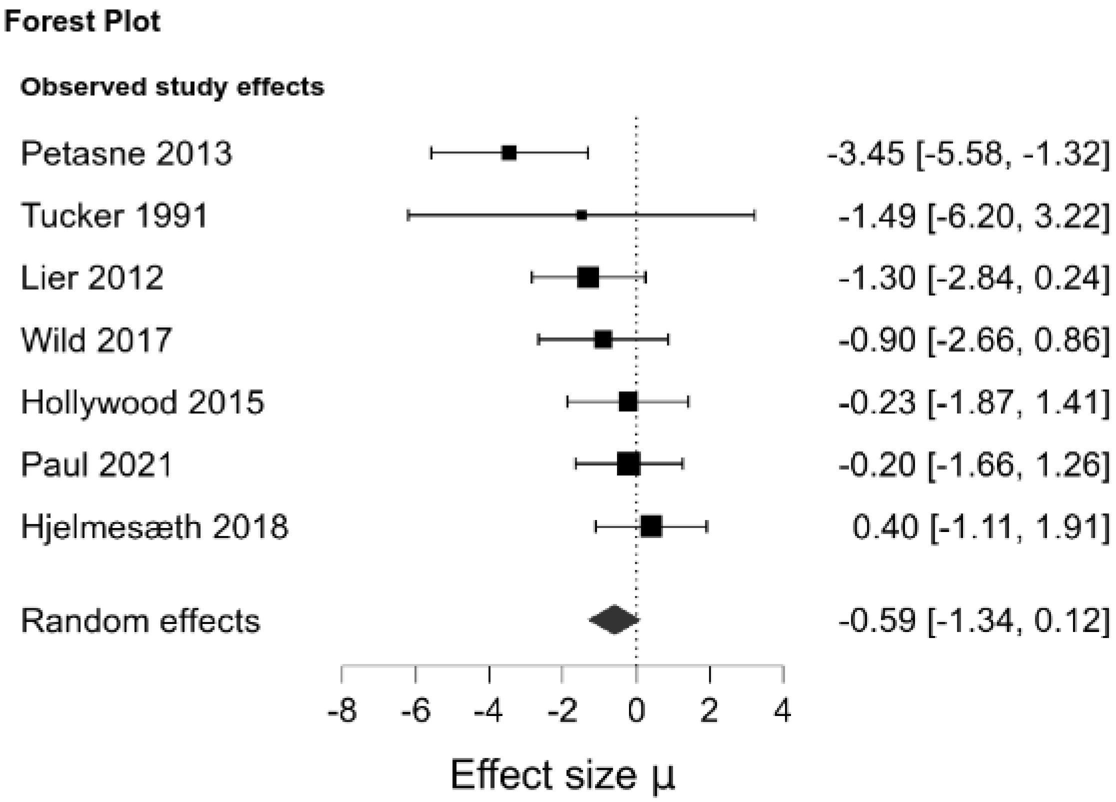

Supplementary Materials, Table S6: Results from random-effects meta-analyses

**Outcome**	**Number of studies**	**Number of participants**	**Mean Difference (95% CrI)**	**Heterogeneity [tau] (95% CrI)**
Change in BMI [kg/m2] 6-12 months follow-up	2	176	-0.29 [-1.6, 0.83]	0.35 (0.04 – 1.74)
Change in BMI [kg/m2] 1-2 years follow-up	7	742	-0.59 [-1.34, 0.12]	0.36 (0.04 – 1.37)
Last follow-up of change in BMI [kg/m2]	7	677	-0.58 [-1.32, 0.15]	0.35 (0.04 – 1.41)
WL [kg] 6-12 months of follow-up	4	416	0.14 [-1.43, 1.97]	0.35 (0.04 – 1.68)
WL [kg] 1-2 years follow-up	8	842	-0.56 [-2.20, 0.66]	0.41 (0.04 – 2.05)
Last follow-up of WL [kg]	9	731	-0.50 [-2.21, 0.77]	0.64 (0.04 – 3.73)
WL [%] 6-12 months follow-up	1	143	-1.60 [-4.68, 1.48]	NA
WL [%] 1-2 years follow-up	2	223	-0.54 [-2.79, 1.07]	0.41 (0.04 – 2.14)
Last follow-up WL [%]	2	204	-1.06 [-4.53, 0.92]	0.49 (0.04 – 2.79)
CrI – credible interval, NA - not applicable, WL – weight loss				

Supplementary Materials, Figure S5: Comparison between psychological intervention versus any control in the WL at 6-12 months follow-up

Posterior Estimates per Model

				95% Credible Interval		
		Mean	SD	Lower	Upper	BF_{01}
Random effects	μ	0.140	0.811	−1.429	1.968	1.443
	τ	0.351	0.490	0.039	1.684	1.090[a]

Note. μ and τ are the group-level effect size and standard deviation, respectively.

[a] Bayes factor of the fixed effects H_1 over the random effects H_1.

Forest Plot ▼

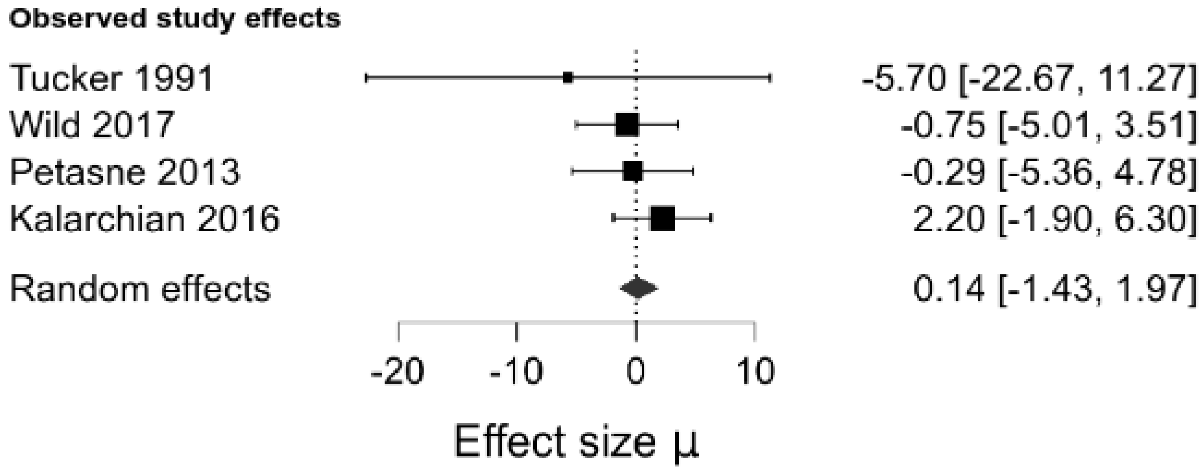

Supplementary Materials, Figure S6: Comparison between psychological intervention versus any control in the WL at 1-2 years follow-up

Posterior Estimates per Model

				95% Credible Interval		
		Mean	SD	Lower	Upper	BF_{01}
Random effects	μ	−0.558	0.715	−2.195	0.656	1.180
	τ	0.412	0.572	0.039	2.052	1.046[a]

Note. μ and τ are the group-level effect size and standard deviation, respectively.
[a] Bayes factor of the fixed effects H_1 over the random effects H_1.

Forest Plot

Observed study effects

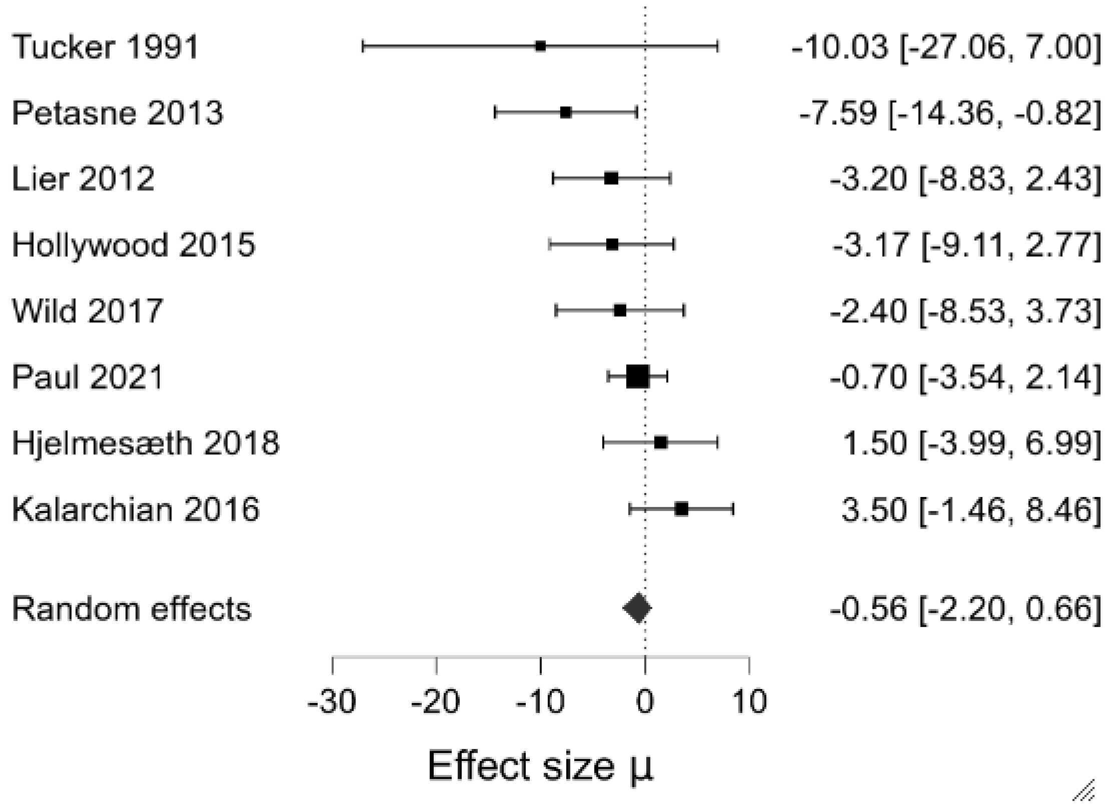

Supplementary Materials, Table S7: **Psychosocial outcomes**

Change in self-efficacy

Study ID	Follow-up	Experimental			Control group			Mean effect [95% CrI]	Tool	Score		Comments
		Mean	SD	Total	Mean	SD	Total			Min	Max	
Wild 2017	Baseline	29.7	5.2	58	29.5	5.2	56	0.20 [-1.71, 2.11]	GSE	0	40	Higher units indicate higher general self-efficacy values
	6 months after surgery	31.6	9.14	49	31.2	8.23	48	0.40 [-2.79, 3.59]				
	12 months after surgery	32.4	6.85	56	33.6	5.99	54	-1.20 [-3.56, 1.16]				
	37.9 months after surgery	33	5.62	39	30.3	5.32	35	2.70 [0.21, 5.19]				
GSE - General Self-efficacy												

Change in quality of life

Study ID	Follow-up	Experimental			Control group			Mean effect [95% CrI]	Tool	Score		Comments
		Mean	SD	Total	Mean	SD	Total			Min	Max	
Paul 2021	Baseline	12.5	2.9	NR	13.3	2.8	NR	-0.8 (for total 125 pts)	WHOQoL-BREF	4	20	Physical health. Higher units indicate higher quality of life
	12 months after surgery	15.1	2.8	NR	15.3	3.0	NR	-0.2 (for total 108 pts)				
	Baseline	13.4	2.4	NR	14.6	2.0	NR	-1.2 (for total 125 pts)		4	20	Psychological health. Higher units indicate higher quality of life
	12 months after surgery	15.0	2.3	NR	15.2	2.7	NR	-0.2 (for total 108 pts)				
	Baseline	13.5	2.3	NR	14.6	2.4	NR	-1.1 (for total 125 pts)		4	20	Social relations. Higher units indicate higher quality of life
	12 months after surgery	14.3	2.8	NR	14.5	3.7	NR	-0.2 (for total 108 pts)				
	Baseline	15.0	1.9	NR	15.4	2.3	NR	-0.4 (for total 125 pts)		4	20	Environment. Higher units indicate higher quality of life
	12 months after surgery	15.6	2.2	NR	15.5	3.1	NR	0.1 (for total 108 pts)				
Wild 2017	Baseline	47.3	19.8	58	49	17.5	56	-1.70 [-8.55, 5.15]	SF-36	0	100	Higher units indicate higher quality of life
	6 months after surgery	70	30.46	49	69.4	27.69	48	0.60 [-10.08, 11.28]				

	12 months after surgery	79.2	30.46	56	75.8	26.19	54	3.40 [-7.02, 13.82]				
	37.9 months after surgery	60.3	16.86	39	62.1	16.57	35	-1.80 [-9.42, 5.82]				
Hollywood 2015	Baseline	2.11	0.57	38	1.96	0.58	32	0.15 [-0.12, 0.42]	SF-36, ADL	0	10	Higher units indicate major problems with ADLs
	12 months after surgery	1.50	0.58	38	1.62	0.71	32	-0.12 [-0.43, 0.19]				
	Baseline	3.96	0.77	38	4.22	0.58	32	-0.26 [-0.58, 0.06]	SEIQoL	1	10	Higher units indicate higher quality of life
	12 months after surgery	4.38	0.51	38	4.20	0.78	32	0.18 [-0.14, 0.50]				
	Baseline	2.5	0.9	38	2.3	0.8	32	0.20 [-0.20, 0.60]	Single item health status scale	0	∞	Odds ratio. Higher units indicate better self-rated health
	12 months after surgery	3.8	0.6	38	3.4	1.1	32	0.40 [-0.03, 0.83]				
SF-36 - Short Form Health Survey, SEIQoL - Schedule for Evaluation of Individualised Quality of Life, ADL - Activities of Daily Living, WHOQoL-BREF - World Health Organization Quality of Life, pts – participants, NR – not reported												

Assessment of 'non-normative' eating patterns

Study ID	Follow-up	Experimental			Control group			Mean effect [95% CrI]	Tool	Score		Comments
		Mean	SD	Total	Mean	SD	Total			Min	Max	
Wild 2017	Baseline	9.8	3.4	58	10	3.7	56	-0.20 [-1.51, 1.11]	EDE	0	168	Higher scores indicate higher level of symptoms
	12 months after surgery	1.4	0.76	56	1.3	0.75	54	0.10 [-0.18, 0.38]				
	37.9 months after surgery	1.65	1.87	39	1.75	1.77	35	-0.10 [-0.93, 0.73]				
Hollywood 2015	Baseline	2.6	0.9	38	2.8	0.7	32	-0.20 [-0.58, 0.18]	DEBQ, emotional eating	1	13	Higher scores indicate higher levels of emotional eating
	12 months after surgery	1.6	0.5	38	1.9	0.7	32	-0.30 [-0.59, -0.01]				
Paul 2021	Baseline	28.4	5.0	NR	27.3	5.7	NR	1.1 (for total 124 pts)	DEBQ external eating	11	55	Higher scores indicate higher levels of external eating
	12 months after surgery	31.4	7.7	NR	30.7	7.3	NR	0.7 (for total 106 pts)				
	Baseline	32.4	6.1	NR	31.0	6.2	NR	1.4 (for total 124 pts)	DEBQ restrained eating	11	55	Higher scores indicate higher levels of restrained eating
	12 months after surgery	23.0	5.2	NR	23.4	5.2	NR	-0.4 (for total 106 pts)				
	Baseline	31.6	10.4	NR	26.5	8.3	NR	5.1 (for total 124 pts)	DEBQ emotional eating	11	55	Higher scores indicate higher levels of emotional eating

	12 months after surgery	25.9	9.6	NR	23.6	9.4	NR	2.3 (for total 106 pts)				
	Baseline	2.5	0.9	NR	2.4	0.9	NR	0.1 (for total 126 pts)	EDE total score	0	132	Higher scores indicate more severe eating disorder
	12 months after surgery	1.3	0.9	NR	1.5	1.0	NR	-0.2 (for total 109 pts)				
Hjelmesæth 2018	Baseline	30.5	18.6	48	49	18.3	50	-18.50 [-25.81, -11.19]	TFEQ-R-21, emotional eating	0	100	The level of dysfunction is indicated by higher scores.
	1 year after surgery	21.4	18.61	42	28.6	17.95	38	-7.20 [-15.22, 0.82]				
	4 years after surgery	40.2	17.79	28	34.3	17.77	33	5.90 [-3.05, 14.85]				
	Baseline	30.4	13.78	48	48.6	13.72	50	-18.20 [-23.65, -12.75]	TFEQ-R-21, uncontrolled eating	0	100	The level of dysfunction is indicated by higher scores.
	1 year after surgery	17.8	13.8	42	23.3	13.39	38	-5.50 [-11.46, 0.46]				
	4 years after surgery	30	12.89	28	27.5	13.25	33	2.50 [-4.08, 9.08]				
	Baseline	68.9	18.25	48	48.7	17.95	50	20.20 [13.03, 27.37]	TFEQ-R-21, cognitive restraint of eating	0	100	The level of dysfunction is indicated by higher scores.
	1 year after surgery	62.6	18.29	42	56.7	17.65	38	5.90 [-1.98, 13.78]				
	4 years after surgery	58.5	17.28	28	52.4	17.49	33	6.10 [-2.65, 14.85]				
Kalarchian 2016	Baseline	4.8	19.7	103	3.1	12.2	84	1.70 [-2.91, 6.31]	SBEs	0	∞	Higher scores indicate more episodes
	6 months after surgery	0.7	2.9	103	1.0	3.8	84	-0.30 [-1.29, 0.69]				
	Baseline	7.9	20.2	103	7.7	18.2	84	0.20 [-5.31, 5.71]	OBEs	0	∞	Higher scores indicate more episodes
	6 months after surgery	0.7	3.0	103	1.3	4.2	84	-0.60 [-1.67, 0.47]				
	Baseline	70.7	10.6	103	70.1	9.7	84	0.60 [-2.31, 3.51]	EBI	26	130	Higher scores indicate higher level of symptoms
	6 months after surgery	89.2	12.2	103	81.5	10.6	84	7.70 [4.43, 10.97]				

DEBQ - The dutch eating behaviour questionnaire, EDE - Eating Disorder Examination, TFEQ - Three Factor Eating Questionnaire, SBE - Subjective bulimic episodes past 28 days on EDE, OBE - Objective bulimic episodes past 28 days on EDE, EBI - Eating Behavior Inventory score.

Change in psychological symptoms

Study ID	Follow-up	Experimental			Control group			Mean effect [95% CrI]	Tool	Score		Comments
		Mean	SD	Total	Mean	SD	Total			Min	Max	
	Baseline	10	5.9	58	8.9	6.1	56	1.10 [-1.10, 3.30]	Depression symptoms PHQ-9	0	27	Asks for cognitive, affective, and somatic depression symptoms

Wild 2017	6 months after surgery	6	9.14	49	6.9	8.23	48	-0.90 [-4.09, 2.29]				
	12 months after surgery	4.1	6.85	56	5.3	5.99	54	-1.20 [-3.56, 1.16]				
	37.9 months after surgery	5.8	4.37	39	8.2	4.73	35	-2.40 [-4.48, -0.32]				
Hollywood 2015	Baseline	2.8	0.9	38	2.9	0.8	32	-0.10 [-0.50, 0.30]	POMS, anxiety	0	36	Higher scores indicate greater anxiety symptoms
	12 months after surgery	2.2	0.9	38	2.4	0.9	32	-0.20 [-0.62, 0.22]				
	Baseline	2.32	1.15	38	2.09	0.93	32	0.23 [-0.26, 0.72]	POMS, depression	0	60	Higher scores indicate a greater number of depressive symptoms
	12 months after surgery	1.48	0.7	38	1.81	0.8	32	-0.33 [-0.69, 0.03]				
Hjelmesæth 2018	Baseline	4.9	2.76	48	7.3	2.82	50	-2.40 [-3.50, -1.30]	HADS, anxiety	0	21	Higher units indicate more probable impairment due to anxiety
	1 year after surgery	4.4	2.57	42	6.2	2.74	38	-1.80 [-2.97, -0.63]				
	4 years after surgery	6.8	2.58	28	5.8	2.54	33	1.00 [-0.29, 2.29]				
	Baseline	2.5	3.1	48	5.0	2.82	50	-2.50 [-3.67, -1.33]	HADS, depression	0	21	Higher units indicate more probable impairment due to depression
	1 year after surgery	1.5	1.93	42	2.0	1.83	38	-0.50 [-1.32, 0.32]				
	4 years after surgery	3.4	2.58	28	1.9	2.54	33	1.50 [0.21, 2.79]				
Kalarchian 2016	Baseline	15.2	10.3	103	14.0	8.9	84	1.20 [-1.55, 3.95]	BDI	0	63	Higher scores indicate a greater number of depressive symptoms
	6 months after surgery	11.8	8.8	103	11.0	8.7	84	0.80 [-1.72, 3.32]				
Petasne 2013	Baseline	54.2%	-	72	58.3%	-	72	0.84 [0.44, 1.63]†	BDI-II	0	100%	Percentage of participants who were experiencing depressive symptomatology in BDI-II.
	6 months after surgery	41.7%	-	72	34.5%	-	72	1.19 [0.61, 2.32]†				
	12 months after surgery	14.9%	-	67	31.8%	-	66	0.38 [0.16, 0.88][a]				
Paul 2021	Baseline	6.3	4.4	NR	4.8	3.0	NR	1.5	QIDS	0	48	Higher scores indicate a greater number of depressive symptoms
	12 months after surgery	4.6	3.7	NR	4.6	3.3	NR	0				

†- odds ratio; PHQ-9 - Patient Health Questionnaire, HADS - Hospital Anxiety and Depression Scale, BDI - Beck Depression Inventory score, BSI - Brief Symptom Inventory, POMS - Profile of Mood States, QIDS - Quick Inventory of Depressive Symptomatology

Supplementary Materials, Figure S7: Subgroup analyses

a) Comparison between psychological intervention versus any control in the change of BMI at 1-2 years follow-up – mixed procedures

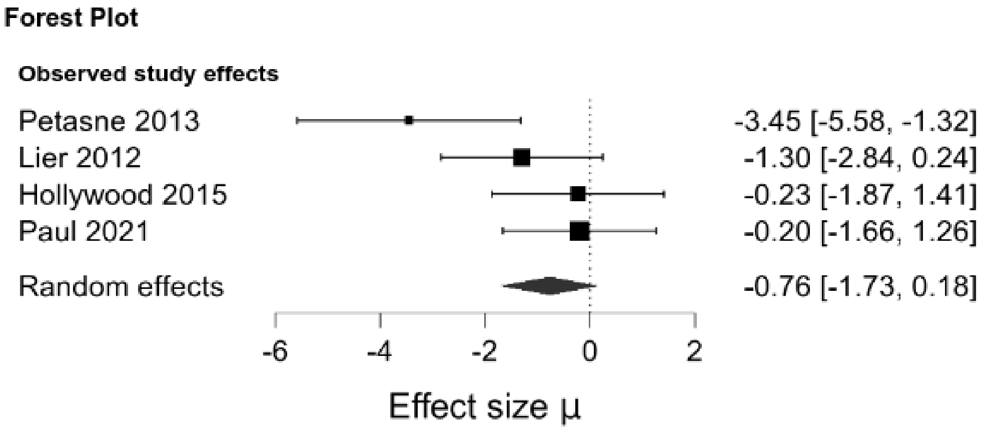

b) Comparison between psychological intervention versus any control in the change of BMI at 1-2 years follow-up– restrictive and mixed procedures

Posterior Estimates per Model

				95% Credible Interval		
		Mean	SD	Lower	Upper	BF_{01}
Random effects	μ	−0.136	0.494	−1.118	0.804	1.975
	τ	0.309	0.390	0.039	1.345	1.145[a]

Note. μ and τ are the group-level effect size and standard deviation, respectively.
[a] Bayes factor of the fixed effects H_1 over the random effects H_1.

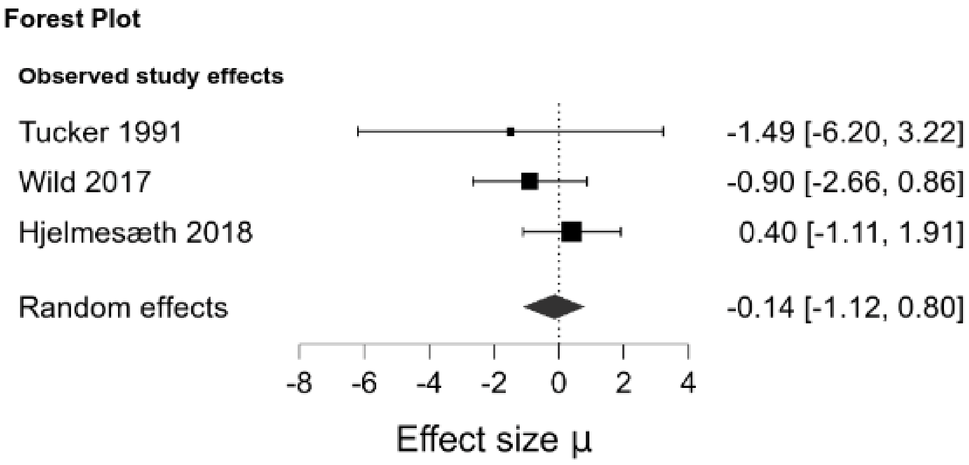

c) Comparison between psychological intervention versus any control in the change of BMI at the last follow-up – mixed procedures

Posterior Estimates per Model

				95% Credible Interval		
		Mean	SD	Lower	Upper	BF_{01}
Random effects	μ	−0.759	0.482	−1.726	0.204	0.460
	τ	0.446	0.561	0.043	1.997	0.913[a]

Note. μ and τ are the group-level effect size and standard deviation, respectively.
[a] Bayes factor of the fixed effects H_1 over the random effects H_1.

Forest Plot

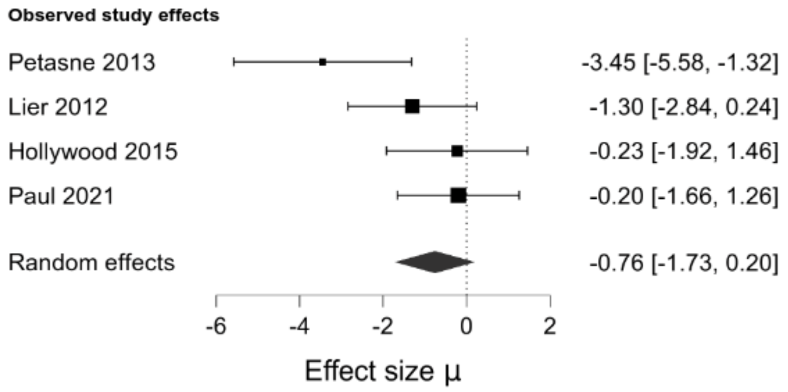

d) Comparison between psychological intervention versus any control in the change of BMI at the last follow-up – restrictive and mixed procedures

Posterior Estimates per Model

				95% Credible Interval		
		Mean	SD	Lower	Upper	BF_{01}
Random effects	μ	−0.058	0.507	−1.098	0.950	1.910
	τ	0.275	0.312	0.039	1.157	1.191[a]

Note. μ and τ are the group-level effect size and standard deviation, respectively.
[a] Bayes factor of the fixed effects H_1 over the random effects H_1.

Forest Plot ▼

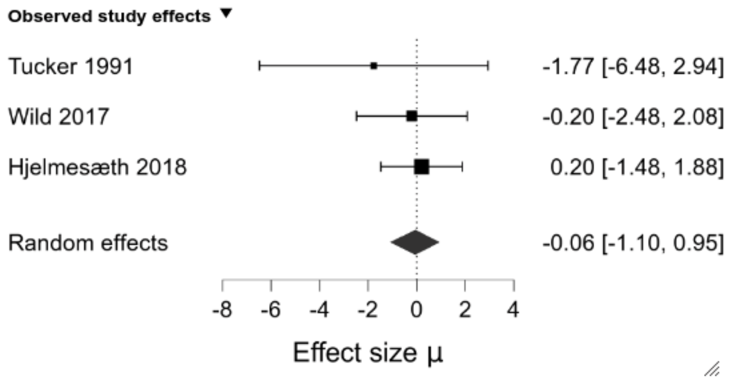

e) Comparison between psychological intervention versus any control in the WL at 1-2 years follow-up– mixed procedures

Posterior Estimates per Model

				95% Credible Interval		
		Mean	SD	Lower	Upper	BF_{01}
Random effects	μ	−1.088	1.025	−3.373	0.472	0.699
	τ	0.438	0.746	0.040	2.501	1.052[a]

Note. μ and τ are the group-level effect size and standard deviation, respectively.
[a] Bayes factor of the fixed effects H_1 over the random effects H_1.

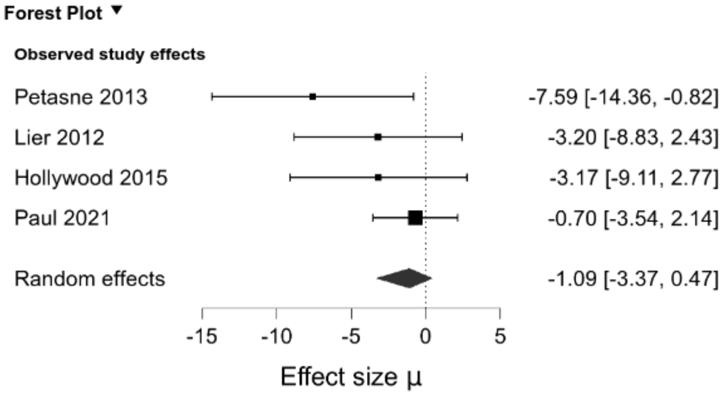

f) Comparison between psychological intervention versus any control in the WL at 1-2 years follow-up– restrictive and mixed procedures

Posterior Estimates per Model

				95% Credible Interval		
		Mean	SD	Lower	Upper	BF_{01}
Random effects	μ	0.259	0.935	−1.478	2.533	1.313
	τ	0.436	0.707	0.040	2.414	1.040[a]

Note. μ and τ are the group-level effect size and standard deviation, respectively.
[a] Bayes factor of the fixed effects H_1 over the random effects H_1.

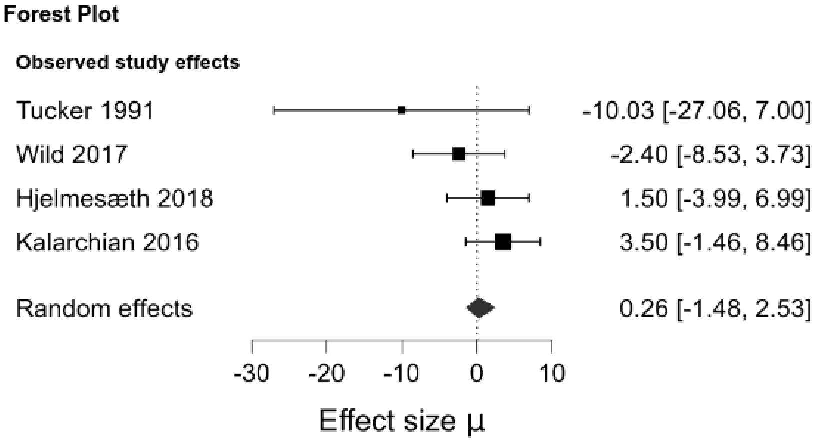

g) Comparison between psychological intervention versus any control in the WL at the last follow-up– mixed procedures

Posterior Estimates per Model

				95% Credible Interval		
		Mean	SD	Lower	Upper	BF_{01}
Random effects	μ	−1.346	1.180	−3.967	0.439	0.579
	τ	0.524	0.904	0.041	3.246	1.002[a]

Note. μ and τ are the group-level effect size and standard deviation, respectively.
[a] Bayes factor of the fixed effects H_1 over the random effects H_1.

Forest Plot

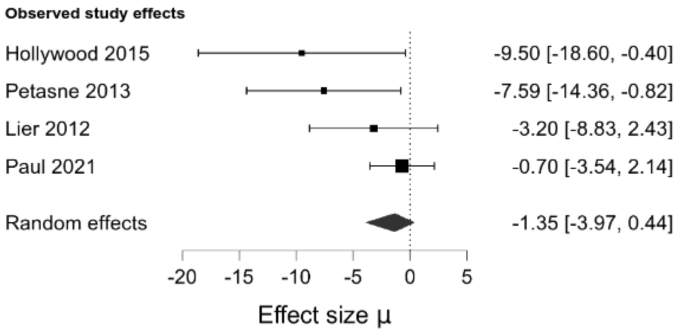

h) Comparison between psychological intervention versus any control in the WL at the last follow-up– restrictive and mixed procedures

Posterior Estimates per Model

				95% Credible Interval		
		Mean	SD	Lower	Upper	BF_{01}
Random effects	μ	0.502	1.016	−1.168	3.023	1.180
	τ	0.518	0.945	0.040	2.998	1.006[a]

Note. μ and τ are the group-level effect size and standard deviation, respectively.
[a] Bayes factor of the fixed effects H_1 over the random effects H_1.

Forest Plot

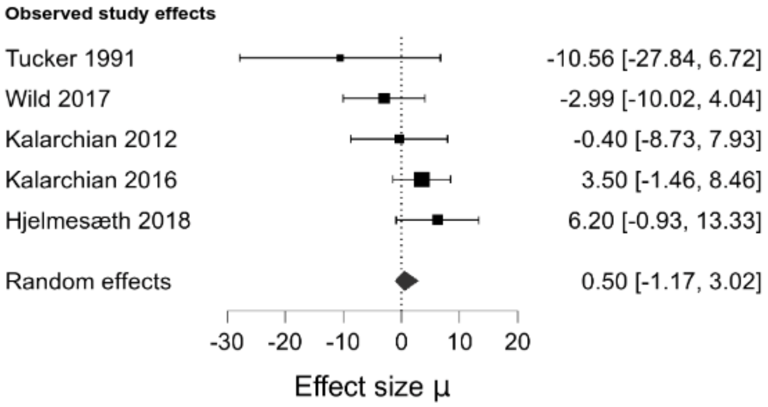

Supplement: Supplementary file 1 [file nutrients-14-01592-s001.zip › nutrients-1632484-supplementary.pdf]
